# Autoimmune RNA dysregulation and seizures: therapeutic prospects in neuropsychiatric lupus

**DOI:** 10.26508/lsa.202201496

**Published:** 2022-10-13

**Authors:** Ilham A Muslimov, Valerio Berardi, Stacy Stephenson, Ellen M Ginzler, John G Hanly, Henri Tiedge

**Affiliations:** 1 Department of Physiology and Pharmacology, The Robert F Furchgott Center for Neural and Behavioral Science, State University of New York Downstate Health Sciences University, Brooklyn, NY, USA; 2 Division of Comparative Medicine, State University of New York Downstate Health Sciences University, Brooklyn, NY, USA; 3 Department of Medicine, State University of New York Downstate Health Sciences University, Brooklyn, NY, USA; 4 Division of Rheumatology, Department of Medicine, Department of Pathology, Queen Elizabeth II Health Sciences Center and Dalhousie University, Halifax, Canada; 5 Department of Neurology, State University of New York Downstate Health Sciences University, Brooklyn, NY, USA

## Abstract

Lupus autoimmunity frequently presents with neuropsychiatric manifestations, but underlying etiology remains poorly understood. Human brain cytoplasmic 200 RNA (BC200 RNA) is a translational regulator in neuronal synapto-dendritic domains. Here, we show that a BC200 guanosine-adenosine dendritic transport motif is recognized by autoantibodies from a subset of neuropsychiatric lupus patients. These autoantibodies impact BC200 functionality by quasi irreversibly displacing two RNA transport factors from the guanosine-adenosine transport motif. Such anti-BC autoantibodies, which can gain access to brains of neuropsychiatric lupus patients, give rise to clinical manifestations including seizures. To establish causality, naive mice with a permeabilized blood–brain barrier were injected with anti-BC autoantibodies from lupus patients with seizures. Animals so injected developed seizure susceptibility with high mortality. Seizure activity was entirely precluded when animals were injected with lupus anti-BC autoantibodies together with BC200 decoy autoantigen. Seizures are a common clinical manifestation in neuropsychiatric lupus, and our work identifies anti-BC autoantibody activity as a mechanistic cause. The results demonstrate potential utility of BC200 decoys for autoantibody-specific therapeutic interventions in neuropsychiatric lupus.

## Abstract (precis)

Lupus autoantibodies directed at neuroregulatory BC200 RNA cause seizure susceptibility in mice, but sequestration with antigen prevents seizures, indicating utility in therapeutic interventions.

## Introduction

In neurons, regulatory brain cytoplasmic (BC) RNAs implement translational control of gene expression in synapto-dendritic domains (Lee et al, 2020; Eom et al, 2021) in one of several mechanisms that, by regulating local protein synthesis, mediate long-term neuronal plasticity (Job & Eberwine, 2001; Gkogkas et al, 2010; Darnell, 2011; Eom et al, 2021). Two stem-loop domains featuring noncanonical structural motif content are determinants of BC RNA functionality.

Noncanonical C-loop motif structures in BC RNA 3′ stem-loop domains, the RNAs’ business centers, implement translational control by interacting with eukaryotic initiation factors (eIFs) 4A and 4B (Eom et al, 2011, 2014). BC RNA control switches from repressive in the basal default state (Wang et al, 2005; Lin et al, 2008; Eom et al, 2011) to permissive via rapid but temporally limited dephosphorylation of eIF4B after glutamate receptor activation (Eom et al, 2014).

A noncanonical guanosine-adenosine (GA) motif structure, so called because it features G and A residues in non–Watson–Crick base pairs, in the rodent BC1 RNA 5′ stem-loop domain is required for dendritic targeting as part of a dendritic targeting element (DTE). This DTE, the RNA’s transport center, specifies synapto-dendritic delivery through interactions with transport factor heterogeneous nuclear ribonucleoprotein (hnRNP A2) (Muslimov et al, 2006, 2011). In model animals, lack of rodent BC1 RNA, either locally at the synapse or globally cell-wide, gives rise to phenotypic manifestations that include seizure disorder and cognitive impairment (Zhong et al, 2009; Iacoangeli et al, 2017; Muslimov et al, 2018).

The GA motif in the rodent BC1 RNA DTE is targeted by autoimmune antibodies from patients with systemic lupus erythematosus (SLE) (Muslimov et al, 2019). Such antibodies, in the following referred to as SLE anti-BC abs, compete with RNA transport factor hnRNP A2 for binding to the BC1 RNA DTE and thus suppress dendritic delivery of the RNA. SLE anti-BC abs were isolated from sera of lupus patients with significant neuropsychiatric involvement, typically seizure disorder or cognitive dysfunction (Mader et al, 2017; Hanly, 2019). Here, we propose a molecular–cellular dissection of anti-BC RNA autoimmunity mechanisms with the goal of developing tailored and specific treatment interventions for patients with neuropsychiatric SLE (NPSLE).

Current treatments of lupus typically focus on various nonspecific anti-inflammatory or immunosuppressive approaches. Such therapies are not usually directed at neutralization or depletion specifically of disease-causing autoantibodies, and serious adverse effects are therefore not uncommon (Prüss, 2021). For this reason, it is desirable to develop therapeutic interventions that are target-specific and are directed at pathogenic autoantibodies but spare other, beneficial antibodies (Prüss, 2021).

In the case of SLE anti-BC abs, the autoantigen is human BC200 RNA. In this study, we present data to demonstrate utility of interventions directed at the specific neutralization of SLE autoantibodies against human BC200 RNA and its DTE-containing 5′ domain. This work had to take into consideration the fact that rodent BC1 RNA and primate BC200 RNA are not phylogenetic orthologs but have developed through unrelated evolutionary lineages (Martignetti & Brosius, 1993a, 1993b; Iacoangeli & Tiedge, 2013; Eom et al, 2021). Thus, equivalence of structure–function relationships can not a priori be assumed. It was therefore critical that such relationships be experimentally established for human BC200 RNA as the development of target-specific immunotherapy approaches (Prüss, 2021) will rely on an in-depth understanding of the DTE epitope structure, its association with transport factors, and their displacement by SLE autoantibodies.

Here, we functionally dissect the motif structure of the BC200 RNA DTE, its interactions with RNA transport factors hnRNP A2 and Purα, and long-term displacement of such factors from the DTE by SLE anti-BC autoantibodies. We identify strong anti-BC reactivity in CSF of NPSLE patients, results to indicate that these autoantibodies can gain access to the brain. We demonstrate that severe seizure susceptibility is elicited by human NPSLE anti-BC abs in animal models and that this seizure phenotype is completely precluded by application of the BC200 autoantigen. Our work provides proof-of-principle evidence that target-specific therapeutic interventions using BC200 decoys are feasible in the treatment of NPSLE patients with anti-BC reactivity.

## Results

We posit that BC RNA DTEs can become autoantigenic in neuropsychiatric SLE (NPSLE). To develop treatment options, it will be essential to arrive at an in-depth understanding of the human BC200 RNA DTE motif structure and its interactions with RNA transport factors and, as an autoantigenic epitope, with SLE anti-BC abs. We report here that Purα is one such RNA transport factor that is required for BC RNA dendritic delivery. We structurally and functionally dissect the human BC200 RNA DTE and identify an apical GA motif that specifies dendritic targeting. The motif interacts with transport factors hnRNP A2 and Purα, both of which are irreversibly displaced from the BC200 RNA DTE by SLE anti-BC abs. We replicate the seizure phenotype of NPSLE patients in animals by i.v. injection of SLE anti-BC abs into mice with a permeabilized blood–brain barrier (BBB). Significantly, the resulting seizure phenotype is completely precluded by co-application of BC200 RNA or derivatives. We suggest that BC200 decoys, based on the autoantibody-interacting BC200 DTE epitope structure, will be valuable in the autoantibody-specific treatment of NPSLE.

### Purα is required for BC RNA dendritic targeting

To ascertain the role of Purα in dendritic BC RNA targeting, we performed knockdown experiments with neurons in primary culture using siRNA (Eom et al, 2014). After Purα knockdown, dendritic targeting was quantified by RNA transport assays using microinjection methodology with sympathetic neurons in primary culture, as described (Muslimov et al, 2006, 2011, 2014).

**Figure 1.**
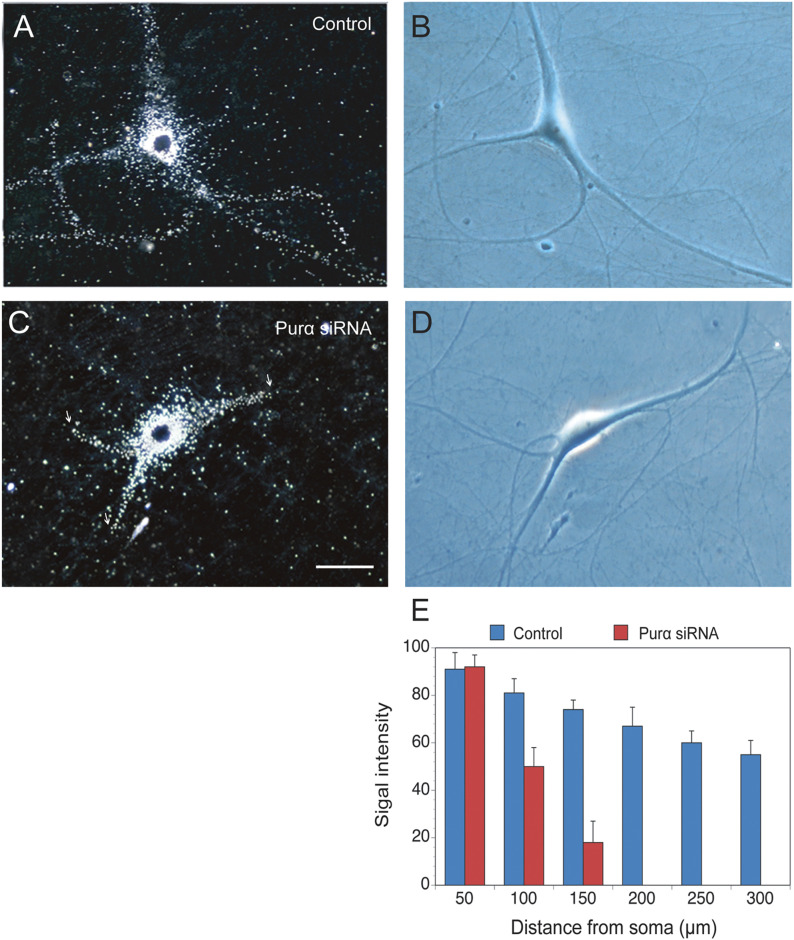
Purα siRNA knockdown: reduction of BC1 RNA dendritic delivery. Microinjection experiments with sympathetic neurons in primary culture were performed with ^35^S-radiolabeled BC1 RNA. **(A, B, C, D)** Dark-field (A, C) and corresponding phase-contrast photomicrographs (B, D) are shown. Labeling signal appears as white silver grains in dark-field photomicrographs. Typical cell morphology and dendritic arborization of sympathetic neurons in primary culture (Muslimov et al, 2006, 2011, 2014) are apparent in phase-contrast photomicrographs. **(A, B)** In sympathetic neurons treated with nontargeting control siRNA, autoradiographic labeling signal along the dendritic extent indicates that microinjected BC1 RNA was distally delivered to dendritic tips. **(C, D)** In cells treated with Purα siRNA, dendritic labeling was reduced in a manner to indicate that microinjected BC1 RNA was transported only into initial dendritic segments. Bar, 50 μm. Arrows indicate extent of dendritic labeling signal. **(E)** Quantitative analysis of relative signal intensities along the dendritic extent. Number of cells/dendrites analyzed: 8/31 for Purα siRNA, 7/29 for nontargeting control siRNA.

We used siRNA directed against the translational start site region of Purα. In sympathetic neurons exposed to nontargeting control (NC) siRNA, dendritic delivery of microinjected ^35^S-labeled BC1 RNA appeared normal as the labeling signal extended along the entire dendritic arborization (Fig 1A and B). In contrast, in sympathetic neurons exposed to Purα siRNA, dendritic delivery was significantly reduced (Fig 1C and D), in comparison with neurons exposed to NC siRNA. Quantitative analysis (Fig 1E) was performed using one-way ANOVA (*P* < 0.0001) and Dunnett’s post hoc test (comparison of dendritic BC1 RNA relative signal intensities after application of Purα siRNA with intensities after application of NC siRNA), *P* > 0.5 for sample points 50 μm, *P* < 0.001 for sample points 100 μm, and *P* < 0.0001 for sample points 150 μm and beyond. Analogous results were obtained when Purα antisense oligonucleotides were used instead of Purα siRNA (not illustrated). The results indicate that Purα knockdown in cultured neurons causes significantly reduced dendritic delivery of BC1 RNA, thus providing evidence that Purα is a BC RNA dendritic transport factor that is required for dendritic delivery.

In regulatory BC RNAs, dendritic targeting competence in DTEs is encoded by architectural motifs that feature noncanonical (non–Watson–Crick, non-WC) purine•purine base pairings (Iacoangeli & Tiedge, 2013; Eom et al, 2021). We hypothesized that such motifs (known as GA motifs because their nucleotide content is exclusively G and A) would interact with Purα (so named because it is a purine-interacting RNA-binding protein). To test this hypothesis, we initially turned to rodent BC1 RNA for which the requisite structural attributes of the DTE have been elaborated in detail (Muslimov et al, 2006, 2011). Indispensable for BC1 RNA DTE functionality, and thus for dendritic transport, is a GA motif in the BC1 RNA 5′ stem-loop domain (Muslimov et al, 2006, 2011). Conversion of the GA motif non-WC base pairings to standard Watson–Crick pairings abolishes dendritic targeting (Muslimov et al, 2006).

**Figure 2.**
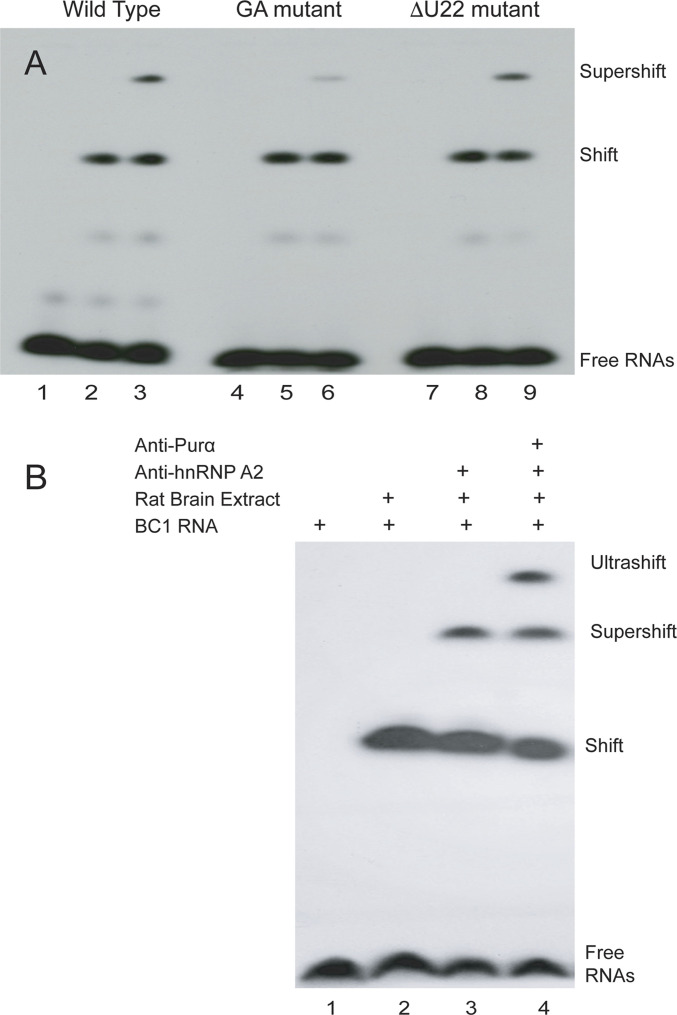
Supershift and ultrashift electrophoretic mobility shift assay (EMSA) assays: interactions of Purα with BC1 RNA. **(A)** Supershift EMSA analysis was performed using native PAGE with radiolabeled wild-type BC1 RNA (lanes 1–3), guanosine-adenosine (GA)–mutant BC1 RNA (lanes 4–6), and ΔU22-mutant BC1 RNA (lanes 7–9) after incubation with brain extract (lanes 2 and 3, 5 and 6, 8 and 9) and with anti-Purα antibodies (lanes 3, 6, and 9). The supershift observed with WT BC1 RNA and ΔU22-mutant BC1 RNA is significantly reduced with GA-mutant BC1 RNA, indicating that an intact GA motif is required for interactions with Purα. **(B)** Ultrashift EMSA analysis demonstrates concurrent binding of Purα and hnRNP A2 to BC1 RNA. Radiolabeled BC1 RNA was loaded in all lanes, rat brain extract in lanes 2–4, anti-hnRNP A2 antibody in lanes 3 and 4, and anti-Purα antibody in lane 4. The ultrashift observed in lane 4 indicates that Purα and hnRNP A2 interact simultaneously with BC1 RNA.

In our work toward elucidation of transport-relevant Purα–BC1 RNA interactions, we performed electrophoretic mobility shift assay (EMSA) supershift assays (Muslimov et al, 2006, 2011) with radiolabeled BC1 RNA in rat brain extracts. As is shown in Fig 2A, incubation of wild-type (WT) BC1 RNA with brain extract caused a shift to lower mobility, whereas in the presence of anti-Purα antibodies, the BC1 RNA signal was shifted further, that is, “supershifted” (Fig 2A). The results reveal that BC1 RNA interacts with Purα in rat brain extracts.

Next, the EMSA supershift experiment was repeated with a BC1 RNA mutant in which the DTE noncanonical GA motif had been abolished by an AAG-to-UCU conversion, resulting in a switch from non-WC base pairings to standard WC base pairings. This GA-mutant BC1 RNA was only negligibly supershifted in the presence of anti-Purα antibodies (Fig 2A). This result indicates that an intact GA motif in the 5′ stem-loop domain is needed for BC1 RNA to interact with Purα. In the final experiment of Fig 2A, the EMSA supershift experiment was conducted with a BC1 RNA mutant in which unpaired U22 had been eliminated (ΔU22 mutant). In this case, the strength of the Purα EMSA supershift was not significantly different from the one observed with WT BC1 RNA.

RNA transport factor hnRNP A2, binding to the BC1 RNA GA motif, is required for dendritic delivery of the RNA (Muslimov et al, 2006, 2011). The fact that Purα binds to the same GA motif (Fig 2A) raises the question whether interactions of the two RNA transport factors with the same motif structure are mutually exclusive or can occur concurrently. We performed supershift-ultrashift EMSA analysis to address this question.

In the experiment shown in Fig 2B, radiolabeled BC1 RNA was incubated with rat brain extract. An antibody specific for hnRNP A2 was added, and a supershift was produced as the antibody interacted with hnRNP A2 in the brain extract. An antibody specific for Purα was then added to the reaction mix, resulting in a further shift to lower mobility (an ultrashift). The anti-Purα antibody now interacted with brain extract Purα that was bound to the BC1 RNA GA motif in a dual immune complex in which an anti-hnRNP A2 antibody interacted with hnRNP A2 that was bound to the same GA motif. These EMSA data indicate that transport factors Purα and hnRNP A2 interact simultaneously with the GA motif in the BC1 RNA 5′ stem-loop DTE, and they are consistent with the notion that concerted transport factor—DTE interactions are requisite for dendritic delivery.

### Human BC200 RNA GA motifs: spatial coding, binding of transport factors, and recognition by NPSLE autoantibodies

Earlier work on regulatory BC RNAs has mostly used rodent BC1 RNA as an experimental model. However, recent data concerning relevance in human disease, in particular in SLE, have motivated a redirection of focus toward human BC200 RNA, necessitating the molecular dissection of its structure–function relationships as a precondition for the development of target-specific therapeutic approaches.

**Figure 3.**
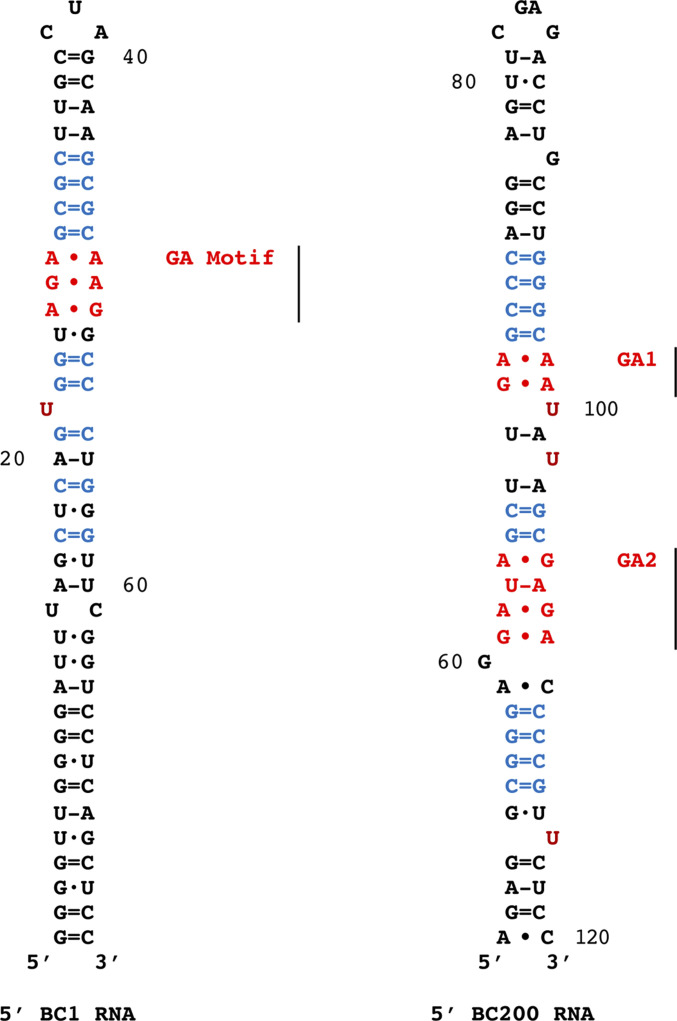
Secondary structure representations: rodent BC1 RNA and primate BC200 RNA 5′ stem-loop domains. See Skryabin et al (1998), Rozhdestvensky et al (2001), Muslimov et al (2006, 2011, 2018), Jung et al (2014), Booy et al (2016, 2018), Lee et al (2020). Noncanonical pairs are symbolized by •, standard Watson–Crick (WC) pairs by = (GC) or - (AU), wobble WC pairs by ·. Noncanonical guanosine-adenosine (GA) core motifs (red) reside in A-form helices that are part of the 5′ stem-loop domains. GA cores are clamped by canonical base pairs which are typically of the GC standard WC type (blue). Unpaired U nucleotides (copper) are located adjacent to GA cores. The BC1 RNA GA motif and the two BC200 RNA GA motifs (GA1 and GA2) are indicated. Noncanonical purine•purine pairs (A•A, A•G, G•A, and G•G) are rather strong, comparable in stability to standard WC pairs (Mladek et al, 2009). Not shown is a pseudoknot in the BC200 RNA 5′ domain, a structure that is not contained in rodent BC1 RNA (see above references). In an alternative BC200 RNA structural model proposal, 5′ and 3′ domains engage in WC base pairing (Sosińska-Zawierucha et al, 2018). This model relies on secondary structure prediction programs that do not take into account contributions of noncanonical base pairings to RNA structure and stability and is not easily compatible with experimental evidence presented in the above references.

In subtypes of neuropsychiatric SLE (NPSLE), patients generate anti-BC RNA autoantibodies (anti-BC abs) that engage human BC200 RNA. After crossing the BBB, anti-BC abs are taken up by neurons where they interfere with BC RNA dendritic delivery (Muslimov et al, 2019). However, whereas the BC1 RNA DTE with its resident GA motif has been scrutinized in detail, little is known about structure and function of the BC200 RNA DTE. Because rodent BC1 RNA and primate BC200 RNA are functional analogs rather than phylogenetic orthologs (i.e., are of separate and independent evolutionary provenance; Martignetti & Brosius, 1993a, 1993b; Iacoangeli & Tiedge, 2013; Eom et al, 2021), differences in function-relevant structural attributes are to be expected. The 5′ stem-loop domains of rat BC1 RNA and human B200 RNA are shown in Fig 3.

Contrasting with rodent BC1 RNA, human BC200 RNA features a 5′ stem-loop domain that contains two putative GA motifs with candidate DTE functionality (apical motif GA1 and basal motif GA2, Fig 3). To examine the functional relevance of these two GA motifs in spatial coding, we converted their noncanonical base pairs to standard WC base pairs, either singly or in combination (see the Materials and Methods section). These BC200 RNA mutants were then tested for dendritic targeting competence and for binding to RNA transport factors.

Dendritic targeting of BC200 RNA mutants was examined using RNA microinjection of neurons in primary culture, as described above (Fig 1). Perikaryal regions of neurons were injected with ^35^S-labeled WT BC200 RNA, with apical GA1-mutant BC200 RNA, with basal GA2-mutant BC200 RNA, or with dual GA1/GA2–mutant BC200 RNA. Progression of radiolabeled RNAs into and along dendritic arborizations was examined over time periods of up to 4 h. During this time, WT BC200 RNA invariably reaches dendritic tips, as has been reported previously (Muslimov et al, 2011).

**Figure 4.**
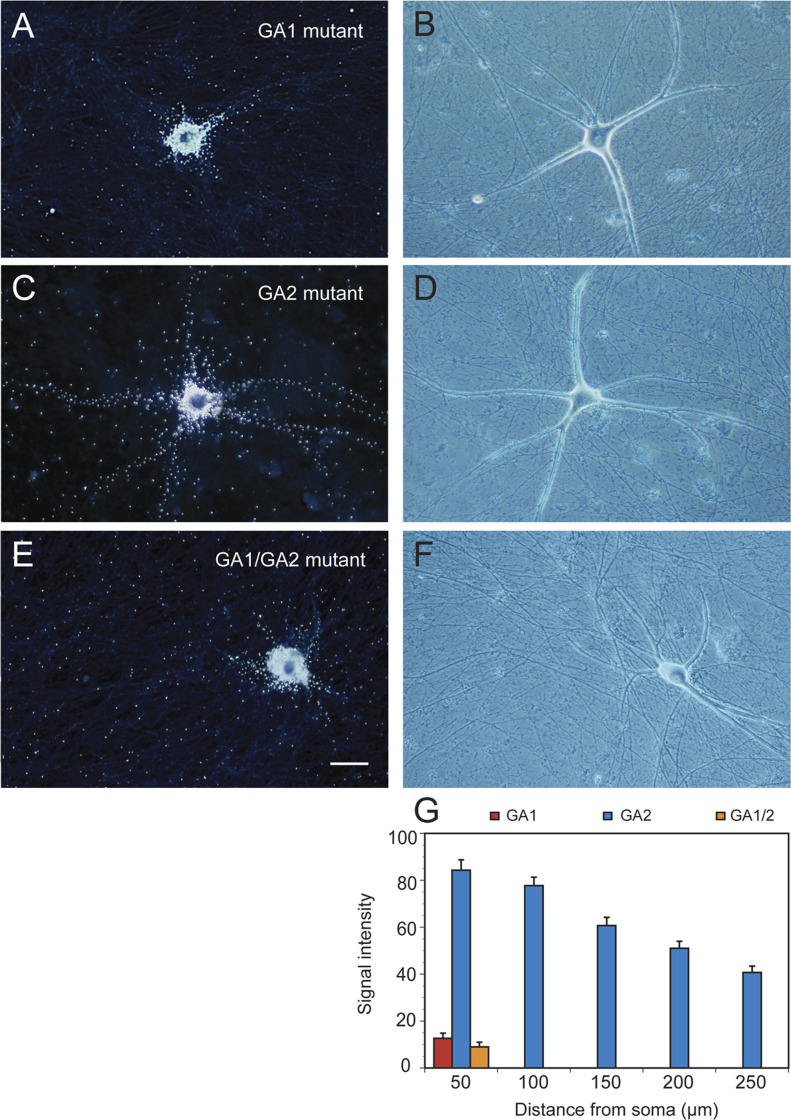
BC200 RNA guanosine-adenosine (GA) motifs: determinants of dendritic targeting competence. **(A, B, C, D, E, F)** Microinjection experiments with sympathetic neurons in primary culture were performed with ^35^S-radiolabeled apical GA1-mutant BC200 RNA (A, B), basal GA2-mutant BC200 RNA (C, D), and GA1/GA2–dual-mutant BC200 RNA (E, F). **(A, B, C, D, E, F)** Dark-field photomicrographs (A, C, E) are accompanied by corresponding phase-contrast photomicrographs (B, D, F). Scale bar, 50 μm. Numbers of cells/dendrites analyzed: A, B, 6/24; C, D, 9/36; E, F, 8/34. **(G)** Quantitative analysis (G) confirmed that apical GA motif GA1, but not basal GA2, is targeting-determinant as dendritic delivery of GA1-mutant BC200 RNA and of GA1/GA2–mutant BC200 RNA, but not that of GA2-mutant BC200 RNA, is significantly reduced.

When GA1-mutant BC200 RNA was microinjected instead of WT BC200 RNA, a strikingly different result was obtained: the labeling signal remained in this case somatically restricted with little or no signal in dendrites except for soma-proximate segments (Fig 4A and B). It is concluded that BC200 RNA carrying a mutant apical GA motif GA1 is lacking dendritic targeting competence. In contrast, BC200 RNA with a mutant basal GA motif GA2 is delivered to dendrites normally (Fig 4C and D), that is, in a manner that is not apparently different from that of WT BC200 RNA (Muslimov et al, 2011). A BC200 RNA derivative lacking both GA motifs GA1 and GA2 was targeting-incompetent, producing a somatic labeling pattern similar to that obtained with GA1-mutant BC200 RNA (Fig 4E and F). Quantitative analysis (Fig 4G) confirmed dendritic targeting competence of GA2-mutant BC200 RNA but lack thereof of GA1-mutant BC200 RNA and GA1/GA2–mutant BC200 RNA: one-way ANOVA (*P* < 0.0001) followed by Dunnett’s multiple comparison analysis (comparison of GA1 mutant with GA2 mutant, *P* < 0.0001 for all interval points; comparison of GA2 mutant with GA1/2 mutant, *P* < 0.0001 for all interval points; comparison of GA1 mutant with GA1/2 mutant, *P* > 0.91 for all interval points). The results indicate that BC200 RNA apical GA motif GA1, but not basal GA2, is required for dendritic targeting.

**Figure 5.**
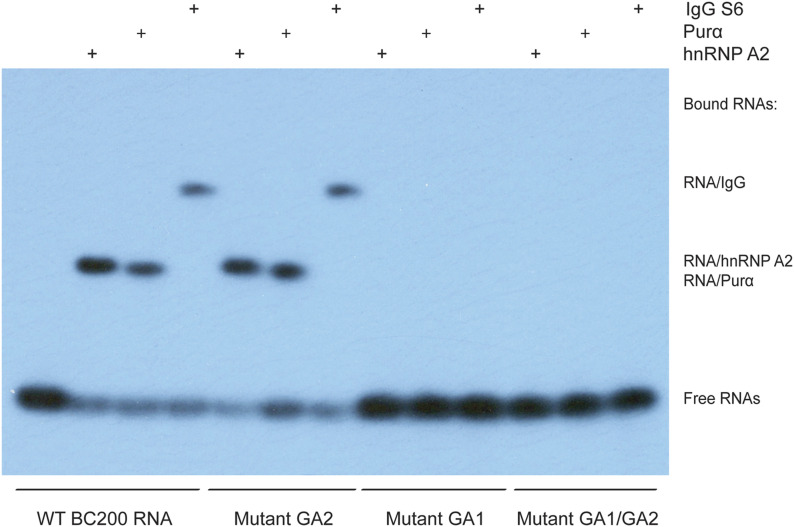
BC200 RNA guanosine-adenosine (GA) motifs: binding of Purα, hnRNP A2, and systemic lupus erythematosus (SLE) anti-BC abs. Electrophoretic mobility shift assay analysis was performed with WT BC200 RNA and mutant BC200 RNAs in which GA motifs GA1, GA2, or both GA1 and GA2 had been eliminated by conversion of noncanonical to standard Watson–Crick base pairings. RNAs were incubated with recombinant Purα, recombinant hnRNP A2, or IgG SLE S6 (purified IgG from an SLE patient with seizures; Muslimov et al, 2019). BC200 RNA with a mutant apical GA1 motif failed to interact with Purα, hnRNP A2, or IgG SLE S6.

The data of Fig 4 predict that RNA transport factors will interact with BC200 apical GA motif GA1 but not with basal GA motif GA2. EMSA results presented in Fig 5 confirm that this is indeed the case. Basal GA2-mutant BC200 RNA was shifted by both hnRNP A2 and Purα in a manner indistinguishable from WT BC200 RNA, indicating that the BC200 RNA GA motif GA2 is not required for interactions of the RNA with any of these two transport factors. In contrast, no mobility shifts were observed when hnRNP A2 or Purα were used with BC200 RNA mutants in which apical GA motif GA1, or GA motif GA1 in conjunction with GA2, had been converted to standard WC base pairings (Fig 5). These results indicate that BC200 RNA apical GA motif GA1 is requisite for binding to both hnRNP A2 and Purα.

The human BC200 RNA apical GA1 motif, but not the basal GA2 motif, shares a structural feature with the rodent BC1 RNA GA motif: a noncanonical A•A/G•A element (Fig 3). The results therefore suggest phylogenetic convergence in the evolution of BC1 and BC200 RNA motif structures. The two RNAs have independently evolved in the rodent and primate lineages, respectively (Martignetti & Brosius, 1993a, 1993b; Skryabin et al, 1998; Rozhdestvensky et al, 2001; Khanam et al, 2007; Iacoangeli & Tiedge, 2013; Eom et al, 2021). While BC1 and BC200 RNAs are thus unrelated in their primary structures (DeChiara & Brosius, 1987; Tiedge et al, 1993), the results of Fig 5 reveal that factor binding-relevant noncanonical A•A/G•A elements are identical, implying evolutionary convergence and analogous functionality.

In the experiments shown in Fig 5, we also tested the ability of SLE anti-BC abs to engage BC200 RNA 5′ GA motif structures. For this work, we used purified IgG from SLE patient S6 (i.e., SLE patient with seizures #6). The results shown in Fig 5 indicate that IgG SLE S6 binds to WT BC200 RNA and to GA2-mutant BC200 RNA but not to GA1-mutant BC200 RNA or GA1/GA2–mutant BC200 RNA. We conclude that binding of IgG SLE S6 to human BC200 RNA relies on the very same BC200 RNA apical A•A/G•A GA motif structure that RNA transport factors hnRNP A2 and Purα interact with. This new structural information will also become important in the design of BC200 decoys for target-specific immunotherapy approaches (Prüss, 2021; also see below and the Discussion section).

EMSA methodology is uniquely suited for the analysis and quantification of interactions between RNAs and proteins (Ryder et al, 2008; Chao et al, 2010). It has been extensively used in the dissection of BC RNA interactions with its binding proteins, including NPSLE autoantibodies (Eom et al, 2011, 2014; Muslimov et al, 2011, 2019). To independently assess interactions of human BC200 RNA with SLE autoantibodies, we applied immunoprecipitation (IP) techniques. Two different IP approaches were used.

**Figure 6.**
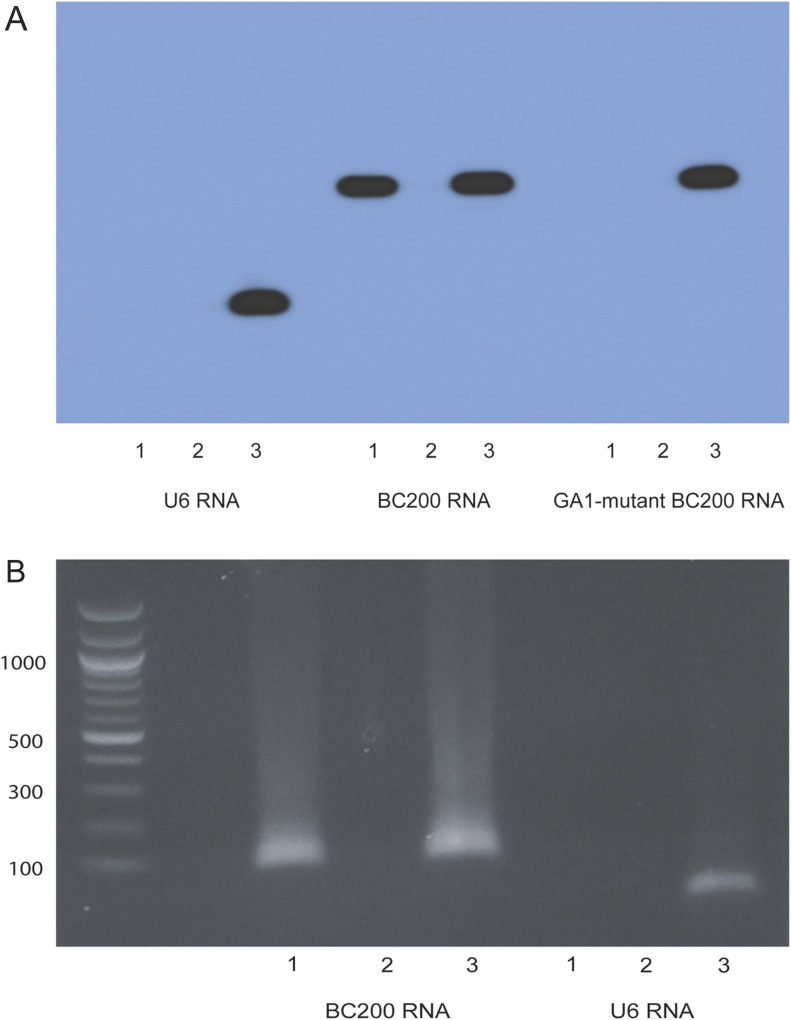
Immunoprecipitation (IP) assays: specificity of systemic lupus erythematosus (SLE) anti BC ab interactions. **(A)** Radiolabeled RNAs were incubated with SLE patient sera after IgG immobilization on Dynabeads Protein G. Three in vitro–transcribed RNAs were used: small nuclear U6 RNA, WT BC200 RNA, and GA1-mutant BC200 RNA. Two patient sera were used: SLE S6 (from an NPSLE patient with seizures) and SLE 24 (from an SLE patient without NP involvement). Lanes were loaded as follows: 1, IP with serum SLE S6; 2, IP with serum SLE 24; 3, input RNA (no IP). A positive IP reaction was observed exclusively after incubation of WT BC200 RNA with SLE serum S6. **(B)** IP was performed with SLE sera S6 and 24 incubated with total RNA isolated from human MCF-7 mammary carcinoma cells. Immunoprecipitated RNAs were amplified by RT–PCR. Two such reactions were run, one with primers specific for BC200 RNA and one with primers specific for small nuclear U6 RNA (see the Materials and Methods section). Lanes were loaded as follows: 1, RT–PCR of IP with serum SLE S6; 2, RT–PCR of IP with serum SLE 24; 3, RT–PCR of input RNA (no IP). Immunoprecipitated BC200 RNA was RT–PCR amplified generating a PCR product with a predicted length of 98 nucleotides. A single product of ∼100 nucleotides was observed in the SLE S6 IP reaction but not in the SLE 24 IP reaction. A second RT–PCR reaction was run to amplify small nuclear U6 RNA, generating a PCR product with a predicted length of 89 nucleotides. No U6 RNA PCR product was observed after IP with either serum SLE S6 or SLE 24. A size ladder was run in the left-most lane, with numbers of nucleotides given in the margin.

In the first IP approach, in vitro–transcribed ^32^P-radiolabeled RNAs were incubated with SLE sera autoantibodies immobilized on Dynabeads Protein G for magnetic separation (So et al, 2016). To scrutinize binding specificity, we used three RNAs with two SLE sera. The three RNAs were (1) WT BC200 RNA, (2) GA1-motif mutant BC200 RNA (which lacks a functional DTE, see Figs 3 and 4), and (3) small nuclear U6 RNA. The two SLE sera were (1) from an NPSLE patient with seizures (SLE S6) and (2) from an SLE patient who had no history of neuropsychiatric manifestations (SLE 24). Fig 6A shows that WT BC200 RNA reacted robustly with, and was thus immunoprecipitated by, serum SLE S6. In contrast, WT BC200 RNA was not immunoprecipitated by serum SLE 24. GA1-motif mutant BC200 RNA or small nuclear U6 RNA was not immunoprecipitated by either serum SLE S6 or serum SLE 24 (Fig 6A). These results indicate that serum SLE S6 specifically interacts with WT BC200 RNA featuring an intact GA motif GA1 but not with a GA1-mutant BC200 RNA or with small nuclear U6 RNA. Serum 24 from a non-neuropsychiatric (non-NP) SLE patient was nonreactive with any of the RNAs tested. Thus, reactivity of SLE S6 with BC200 RNA is highly specific.

In a second type of IP approach, we worked with RNA preparations from cells expressing BC200 RNA. Invasive breast cancer cells are known to express BC200 RNA (Iacoangeli et al, 2004, 2018) as are MCF-7 cells, a breast cancer cell line derived from an invasive ductal mammary carcinoma (Iacoangeli et al, 2018). We isolated total RNA from MCF-7 cells and performed IP with serum SLE S6 or serum SLE 24, followed by RT–PCR with primers specific for BC200 RNA. An IP RT–PCR product was only observed with serum SLE S6, not however with serum SLE 24 (Fig 6B). In contrast, IP followed by RT–PCR with primers specific for small nuclear U6 RNA did not generate an IP PCR product with either serum SLE S6 or serum SLE 24. The results indicate that autoantibodies in the serum from patient SLE S6 specifically interact with BC200 RNA in total RNA isolated from MCF-7 breast cancer cells.

### SLE anti-BC abs: long-term displacement of transport factors from human BC200 RNA

Results presented above (Figs 5 and 6) indicate that SLE anti-BC abs bind to the same apical GA motif (GA1) in the human BC200 RNA 5′ stem-loop domain as do transport factors hnRNP A2 and Purα. As BC RNA binding of hnRNP A2 and Purα is concurrent (Fig 2), the question arises as to what impact SLE anti-BC abs will have on the binding of the two transport factors to BC200 RNA.

We performed long-term EMSA displacement analysis to address this question. In this approach, we incubated radiolabeled BC200 RNA first with SLE anti-BC IgG to allow binding and subsequently added a transport factor (hnRNP A2 or Purα) to examine whether the factor was able to displace the SLE anti-BC IgG from BC200 RNA. Vice versa, in a second set of experiments, radiolabeled BC200 RNA was first incubated with a transport factor (hnRNP A2 or Purα) to allow binding after which an SLE anti-BC IgG was added to the reaction mix to examine its long-term ability to displace the transport factor from BC200 RNA. Transport factors and SLE anti-BC IgGs were used at 1:1 molar ratios. The course of displacement was monitored for a total period of 106 h at 37°C.

**Figure 7.**
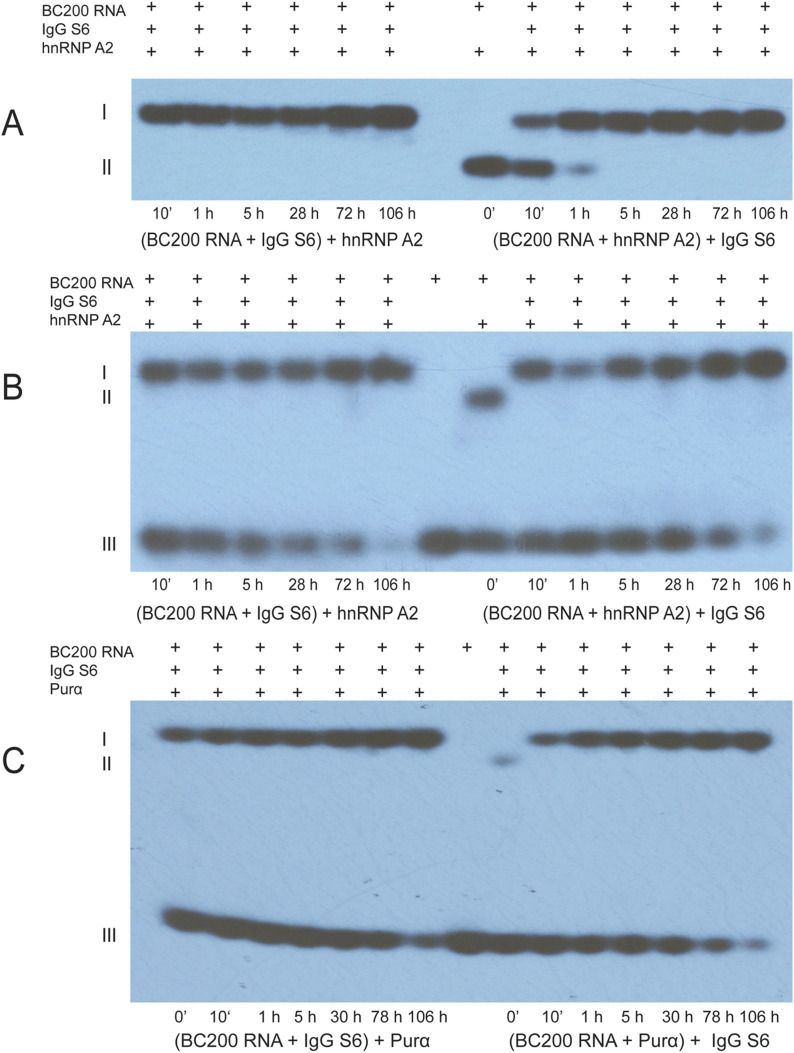
Long-term displacement of transport factors from BC200 RNA by systemic lupus erythematosus (SLE) anti-BC abs. Electrophoretic mobility shift assay displacement analysis was performed with radiolabeled BC200 RNA, transport factors, and IgG SLE S6. **(A, B, C)** In the left parts of the gels shown in (A, B, C), BC200 RNA was first incubated with IgG SLE S6 and subsequently, after 30 min, with a transport factor. **(A, B, C)** In the right parts of the gels shown in (A, B, C), BC200 RNA was first incubated with a transport factor and subsequently, after 30 min, with IgG SLE S6. **(A, B)** Transport factor hnRNP A2 was used; (C) transport factor Purα was used. **(A, B)** I denotes BC200 RNA—IgG SLE S6 complexes, and II denotes BC200 RNA—hnRNP A2 complexes. **(B)** III denotes free (unbound) BC200 RNA (which was allowed to run off the gel in A). **(C)** I denotes BC200 RNA—IgG SLE S6 complexes, II denotes BC200 RNA—Purα complexes, and III denotes free (unbound) BC200 RNA. Note that Purα binds less strongly to BC200 RNA than hnRNP A2.

Working with IgG SLE S6, we observed that this SLE anti-BC ab bound BC200 RNA rapidly and avidly and that it was not displaced from the RNA by transport factor hnRNP A2 over the course of 106 h (>4 d, Fig 7A and B). When conversely hnRNP A2 was first incubated with BC200 RNA, binding was equally rapid (Fig 7A and B). In this case, however, hnRNP A2 was displaced from BC200 RNA by subsequently added IgG SLE S6, a displacement the time course of which varied somewhat between experiments but always reached near-completion within 1 h. SLE anti-BC ab binding to BC200 RNA seems to have reached near-completion after 1 h (although it continued to increase slightly thereafter). A very similar mode of long-term trilateral interactions and displacement involving BC200 RNA, transport factor, and SLE anti-BC ab was observed when Purα was used instead of hnRNP A2 (Fig 7C).

We conclude that SLE anti-BC IgG is able to displace transport factors hnRNP A2 and Purα from human BC200 RNA and to disallow re-engagement of those factors with the RNA over at least several days.

### Neuropsychiatric phenotypic manifestations: correlations and causality

How do SLE anti-BC abs, causing the described molecular–cellular alterations, give rise to phenotypic manifestations? Two approaches were used to address this question. In the first approach, correlative by nature, we worked with SLE patient material. In the second approach, to be detailed in the following section, we worked with animal models to establish causality.

A 24-yr-old male with a diagnosis of SLE (made elsewhere in December 2015, with clinical manifestations including malar rash, oral ulcers, arthritis, and lymphadenopathy) was admitted to University Hospital of Brooklyn (UHB) of SUNY Downstate Health Sciences University (DHSU) in March 2020 with fever, new severe tonic-clonic seizures, new proteinuria, and pancytopenia. Workup to assess the etiology of his seizures included normal brain MRI and MRA and negative assessment for infection including bacterial cultures of serum and CSF, negative CSF cryptococcal antigen, and a negative COVID-19 test. Treatment was initiated with anti-seizure medication, intravenous pulse corticosteroids, and intravenous cyclophosphamide. The patient responded well to treatment, clinical manifestations of active lupus resolved, and steroids were slowly tapered.

The first blood sample was obtained from the patient in March 2020, before treatment was initiated. The second blood sample was obtained during a routine follow-up visit to UHB in May 2021, at that time when the patient had been seizure-free for more than a year.

**Figure 8.**
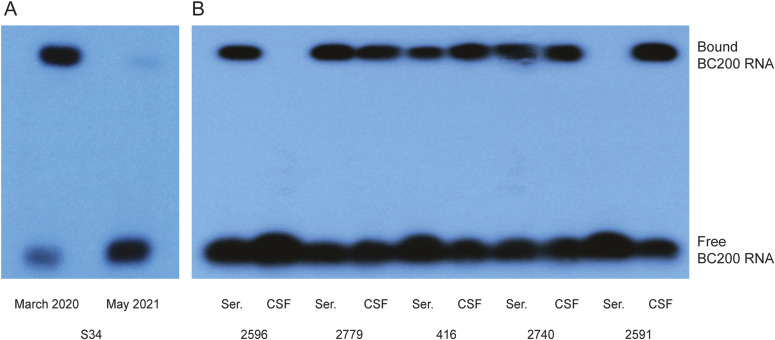
Anti-BC200 autoimmune reactivities in systemic lupus erythematosus (SLE) patient sera and CSF. **(A)** NPSLE patient S34 (m, age 24) was admitted with recurrent severe seizures in March 2020. Electrophoretic mobility shift assay (EMSA) analysis revealed high anti-BC200 reactivity in serum. Patient S34 reported back after having been seizure-free for more than a year. At this time, negligible anti-BC200 reactivity was detected. **(B)** Nova Scotia NPSLE paired patient sera and CSF were examined using EMSA. Differential anti-BC200 CSF versus serum reactivities were apparent as follows. In patient 2596, reactivity was robust in serum but absent from CSF; in patients 2779, 416, and 2740, reactivity was robust in both serum and CSF (serum levels > CSF levels for patient 2779, CSF > serum for patient 416, and serum ≈ CSF for patient 2740); in patient 2591, reactivity was robust in CSF but absent from serum. Data shown represent samples from 5 of a total of 24 examined patients. Results with samples from all 24 patients are provided in Table 1 along with relevant patient data including neuropsychiatric presentation. Patient codes are those of Dalhousie University, Nova Scotia. The EMSA running pattern (i.e., the degree of mobility shift) of BC200 RNA was found invariable no matter whether the RNA had been incubated with SLE IgG, SLE serum, or SLE CSF (see also Fig 7), a result to confirm earlier findings that SLE anti-BC reactivities are attributable to the IgG class of immunoglobulins (Muslimov et al, 2019).

EMSA analysis revealed high serum levels of anti-BC200 reactivity at the time of first admission but barely detectable levels at the time of the follow-up visit (Fig 8A). While the evidence is correlative and from a single patient, the data suggest that high serum levels of SLE anti-BC autoantibodies are linked to severe neuropsychiatric disease.

For SLE anti-BC autoantibodies to cause neuropsychiatric manifestations, they would be expected to have gained at least temporary access to the brain. To test this prediction, we obtained paired samples of serum and CSF from NPSLE patients at the Division of Rheumatology, Department of Medicine, Queen Elizabeth II Health Sciences Center and Dalhousie University. Fig 8B shows EMSA results from five representative NPSLE patients. Patient 2596 exhibited strong anti-BC200 reactivity in serum but no reactivity in CSF, patients 2779, 416, and 2740 had robust reactivity in both serum and CSF (with differential ratios of CSF versus serum reactivities), and patient 2591 had strong reactivity in CSF but no reactivity in serum. The patient 2591 result is remarkable as it raises questions concerning the provenance of the anti-BC200 autoimmune reactivity and the locus of autoantibody synthesis.

Results from a total of 24 SLE patients, including those presented in Fig 8B, are presented in Table 1. Patients in this table are grouped into four categories according to anti-BC200 reactivity levels in serum and CSF. Category 1 patients (lines 1–5) are those with EMSA-established anti-BC200 reactivity in serum but not in CSF. Acute confusion was the predominant neuropsychiatric symptom of these patients. Category 2 patients (lines 6–13) showed anti-BC200 reactivity in both serum and CSF. Seizures were the most common symptom in this patient category; in addition, psychosis and acute confusion were observed. In category 3 (lines 14–16), patients expressed anti-BC200 reactivity in CSF but not in serum. Seizures, along with psychosis and acute confusion, were symptoms in this category. It is noted that CSF anti-BC200 reactivity levels in these patients were the highest within the four categories (i.e., at the high end of the ++ reactivity level of Table 1). Neuropsychiatric symptoms were particularly severe in this category as, for example, patient 1327 presented with highly aggressive neuropsychiatric SLE with seizures at an age of 16 years, and patient 2591 was encephalopathic, responsive but nonverbal on admission to hospital. Both were treated and responded to high-dose IV glucocorticoids and cyclophosphamide. In addition to the category 3 patient data, three unpaired CSF samples with strong anti-BC200 reactivities are listed in lines 17–19.

**Table 1.** Anti-BC200 autoimmune reactivity in serum and CSF samples from 24 NPSLE patients.

Line	Patient code	Age	Sex	EMSA serum	EMSA CSF	NP because of systemic lupus erythematosus	NP not because of systemic lupus erythematosus
1	311	38	F	++		Acute confusion	
2	2,267	13	F	+		Acute confusion, coma	
3	2,272	23	M	+		Psychosis	
4	2,596	29	F	+		Microinfarcts because of APS	
5	2,768	30	F	+		Seizures, acute confusion	
6	1,557	40	F	++	+	Seizures	
7	2,532	43	F	++	+		Depression
8	2,779	34	F	++	+	Acute confusion	
9	2,685	52	F	++	++	Seizures, myelitis	
10	2,686	51	F	++	++	Myelitis	
11	2,740	27	M	++	++	Stroke (APS)	
12	2,827	13	F	++	++	Seizures, coma; concurrent MAS	
13	416	27	F	+	++	Psychosis	Depression
14	700	63	F		++	Seizures, psychosis	Demyelination
15	1,327	16	M		++	Seizures	
16	2,591	41	F		++	Acute confusion, encephalopathy	
17	2,689	26	F	n/a	++	Seizures, stroke (APS)	
18	2,757	27	F	n/a	++	Psychosis	
19	2,777	20	F	n/a	++	Seizures	
20	1,004	51	F			Seizures, coma	
21	1,202	16	F			Seizures	
22	1,636	76	F				Acute confusion
23	2,317	36	M			Seizures	
24	2,616	29	F				

Reactivity levels (−, negative; +, mid to high; ++, very high) were established by EMSA as shown in Fig 7B. APS, antiphospholipid syndrome; MAS, macrophage activation syndrome; MG, myasthenia gravis; MS, multiple sclerosis; NP, neuropsychiatric manifestations. Age refers to age of a patient at onset of NP symptoms. Patient codes are those of Dalhousie University, Nova Scotia.

Patients of category 4 (lines 20–24) are those where no anti-BC200 reactivity was recorded in either serum or CSF. Two of these patients were free of SLE-attributable neuropsychiatric symptoms, whereas the other four had presented with seizures or psychosis. It is concluded that these NP symptoms were caused by reactivities directed at autoimmune antigens other than BC200 RNA.

In summary, the data of Fig 8B and Table 1 indicate that in a subset of NPSLE patients, anti-BC abs are present in CSF and thus have access to the brain. Anti-BC abs may have entered the CSF via a compromised BBB, although an alternative scenario is conceivable in the case of category 3 patients: intrathecal autoantibody synthesis (Prüss, 2021; see also the Discussion section).

### SLE anti-BC abs causing seizure disorder

We will now address the question whether SLE anti-BC abs are causing neuropsychiatric manifestations in lupus. To this end, we worked with an animal model in which naive WT mice were i.v. injected with SLE anti-BC sera or IgGs. Injected animals were inspected for signs of macroscopic or microscopic pathology, in particular brain pathology. None were noted (see also Muslimov et al [2019]).

We recall that model animals in which endogenous BC1 RNA is lacking cell-wide or locally at the synapse are highly seizure-susceptible upon sensory stimulation (Zhong et al, 2009; Muslimov et al, 2018). We therefore asked whether SLE anti-BC abs, obtained from NPSLE patients with seizures, would engender analogous seizure susceptibility upon introduction into animals. I.v. injections were performed following the protocol of the Diamond Laboratory, using LPS to permeabilize the BBB (Kowal et al, 2006). Neurons are known to take up antibodies via clathrin-dependent Fcγ receptor-mediated endocytosis, and autoantibodies so internalized can disrupt intracellular functions (Magrys et al, 2007; Elkon & Casali, 2008; Geis et al, 2010; Congdon et al, 2013; Douglas et al, 2013; Kazim et al, 2017; Rhodes & Isenberg, 2017; Prüss, 2021).

**Figure 9.**
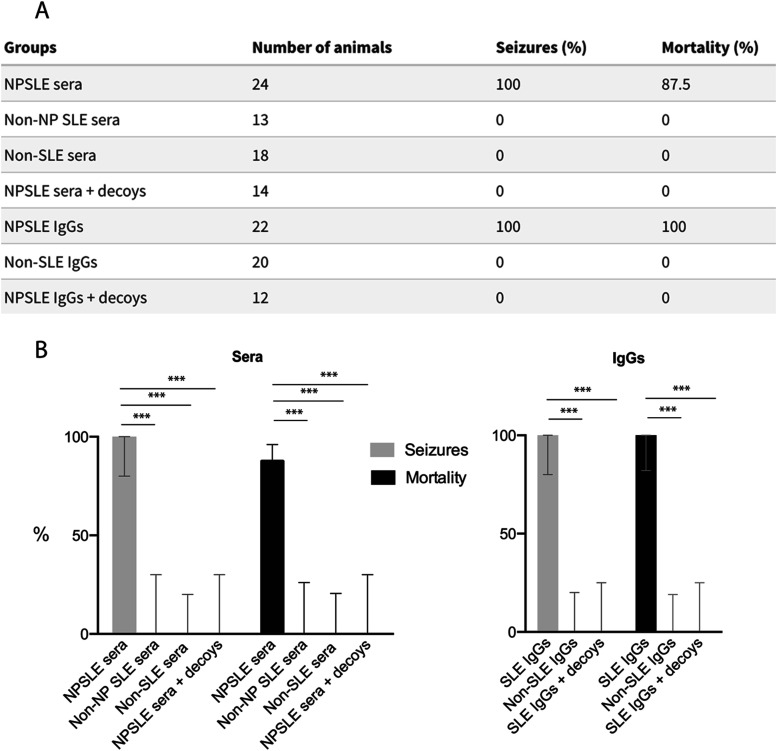
Seizure susceptibility and mortality caused by NPSLE anti-BC200 autoantibodies. Groups are defined as follows. NPSLE sera/IgGs from NPSLE patients S1, S6, S13, and S17; non-NP systemic lupus erythematosus (SLE) serum from patient SLE 10; non-SLE sera/IgGs from patient rheumatoid arthritis 1, from patient multiple sclerosis 1, and from heathy subject 4. N, number of animals. Nine additional animals were injected with saline: none of them seized. Animals in the NPSLE sera/IgGs + decoys group were injected with sera or IgGs together with BC200 decoys. Full-length BC200 RNA (20 animals injected) and the BC200 5′ domain (6 animals injected) were used as decoys, with identical results. **(A)** Original raw data in table format. **(B)** Quantitative and statistical analysis: Fisher’s exact test, Agresti-Coull interval method. 95% confidence intervals are shown.

We first injected WT animals with sera from four different NPSLE patients with seizures (SLE S1, S6, S13, and S17). As controls, we used sera from a healthy subject (HS4) and from patients with non-SLE autoimmune disorders (rheumatoid arthritis [RA], patient RA1, and multiple sclerosis [MS], patient MS1). In a further control, WT mice were injected with serum from an SLE patient without neuropsychiatric manifestations (patient SLE 10); serum from this patient was lacking anti-BC reactivity (Muslimov et al, 2019). When subjected to auditory stimulation on day P19, all animals injected with NPSLE sera succumbed to severe generalized, tonic-clonic seizures (Fig 9A). Mortality from seizures was 87.5%. In contrast, none of the animals injected with serum HS4, RA1, MS1, or non-NP SLE 10 seized or died: Fisher’s exact test, *P* < 0.0001 (Fig 9B).

Seizures were preceded by a brief period of wild running. Qualitatively, wild running and seizures were similar if not identical to the seizure phenotype of BC1 KO animals (Zhong et al, 2009, 2010). However, there were quantitative differences as all SLE serum-injected animals underwent seizures (versus 84% of BC1 KO animals) with a mortality of 87.5% (versus 23% of BC1 KO animals, see also below). It is noted that while auditory exposure was used in our work to assess seizure susceptibility (for reasons given in the Materials and Methods section), incidental observations suggest that other types of sensory stimulation (olfactory, visual) can also trigger seizure activity in BC1 model animals and in BBB-permeabilized WT mice injected with NPSLE anti-BC abs. In the rodent brain, BC1 RNA is prominently expressed in sensory systems, among others (Tiedge et al, 1991; Lin et al, 2001), and we submit that synapto-dendritic lack of the regulatory RNA in such systems can cause hyperexcitability.

We next injected WT animals with purified IgG preparations from NPSLE patients S1, S6, or S13 (Fig 9). These experiments were initially complicated by the fact that after the above injection routine, animals almost immediately seized and died when exposed to moderate environmental noise, for example during transit from their quiet-zone home area (see the Materials and Methods section) to the location of seizure susceptibility testing. We therefore modified the injection routine by omitting the P14 i.v. injection and lowering the amount of SLE IgG injected on day P15 by a factor of 4, as a result reducing the total amount of injected SLE IgG by a factor of 8. With such injection parameters, animals did not seize while in transit. However, upon experimental auditory stimulation, all animals injected with IgG SLE S1, S6, or S13 entered into a short wild running phase which was directly followed by generalized seizures of the tonic-clonic type (Video 1). All animals injected with NPSLE IgGs died while undergoing seizures (Fig 9).

Video 1 Anti-BC RNA NPSLE autoantibodies cause seizure susceptibility. WT animals were i.v. injected with lupus IgG S6 (Video 1) or with lupus IgG S6 in conjunction with BC200 RNA (Video 2). The animal in this video seized and died within a minute of sound exposure. The two animals in both the videos were littermates. Both male and female animals were used in seizure experiments, with identical results in terms of seizure and mortality rates. The videos are in MP4 format. They can be viewed using the VLC media player (download available at https://www.videolan.org/vlc/). Download video

In control experiments, WT animals were i.v. injected with purified IgG preparations HS4, RA1, or MS1. No animal thus injected underwent seizures or died: Fisher’s exact text, *P* < 0.0001 (Fig 9).

The seizure phenotype of animals injected with SLE anti-BC IgGs is qualitatively identical to that of BC1 KO animals (Zhong et al, 2009, 2010) but quantitatively much more severe. The question is thus raised as to why the seizure phenotype of WT animals in which synapto-dendritic presence of native BC1 RNA has been reduced as a result of NPSLE anti-BC ab action is similar to but more severe than that of BC1 KO animals in which the RNA is absent cell-wide. We propose that compensatory changes may play a role in BC1 KO animals in which the RNA has been lacking throughout ontogenetic development but much less so in WT animals injected with SLE anti-BC abs in which case depletion of synapto-dendritic BC1 RNA is acute and occurs within days.

A natural candidate for BC1 compensation would be BC2 RNA, a neuronally expressed paralog of BC1 RNA (McKinnon et al, 1987; Skryabin et al, 2003). BC2 RNA is shorter than BC1 RNA and is sequence-homologous to BC1 nucleotides 1 to about 110 (Sutcliffe et al, 1984; Skryabin et al, 2003). Thus, BC2 RNA contains the 5′ stem-loop domain that interacts with transport factors, part of the A-rich central region, but not the 3′ stem-loop domain that interacts with eIF 4B (Eom et al, 2014). BC2 RNA translational control competence would therefore be limited to interactions with poly(A)-binding protein and possibly eIF 4A (Lin et al, 2008; Eom et al, 2011). BC2 RNA is expressed in brains of BC1 KO animals (Skryabin et al, 2003), raising the possibility that it may be able to partially compensate for the absence of BC1 RNA.

We i.v. injected BC1 KO mice with SLE IgG S6 in combination with BBB permeabilization and scored the animals for seizure susceptibility as described above. All animals underwent tonic-clonic seizures with a mortality of 100% (N = 6). The respective numbers for non-injected BC1 KO animals are 84% (seizure rate) and 23% (mortality) (Zhong et al, 2009). The results are consistent with the notion that in BC1 KO animals, BC2 RNA is partially compensating for the lack of BC1 RNA but that the action of SLE anti-BC abs blocks such compensation, presumably by inhibition of BC2 RNA synapto-dendritic delivery. It is noted that this scenario is rodent-specific as a BC2-equivalent paralog of BC200 RNA does not appear to exist in humans (Martignetti & Brosius, 1993a).

In summary, our data show that SLE anti-BC IgGs from NPSLE patients with seizures are highly potent epileptogenic agents when introduced into WT animals with a permeabilized BBB. However, a key question remains to be addressed. Can the seizure phenotype that is elicited by SLE anti-BC abs be prevented by sequestration of autoantibodies with antigen or derivatives? We used BC200 RNA and the BC200 5′ domain as decoys, testing their efficiencies in engaging and neutralizing SLE anti-BC abs in vivo. Anti-BC autoantibodies, in the form of SLE patient sera or purified IgG, were i.v. coinjected with BC200 decoys into naive WT mice, in conjunction with LPS i.p. injection for BBB permeabilization. Mice so injected never seized or showed any indication of seizures (Fig 9 and Video 2). Mortality from seizures was recorded at 0%. It is concluded that the phenotype rescue efficiency of BC200 decoys was 100% for animals injected with SLE sera or purified SLE IgGs (Fig 9B; Fisher’s exact text, *P* < 0.0001).

Video 2 Anti-BC RNA NPSLE autoantibodies cause seizure susceptibility. WT animals were i.v. injected with lupus IgG S6 (Video 1) or with lupus IgG S6 in conjunction with BC200 RNA (Video 2). The animal in this video did not seize within a 15-min time period of auditory stimulation. The two animals in both the videos were littermates. Both male and female animals were used in seizure experiments, with identical results in terms of seizure and mortality rates. The videos are in MP4 format. They can be viewed using the VLC media player (download available at https://www.videolan.org/vlc/). Download video

The above rescue data show that the seizure phenotype elicited by NPSLE autoantibodies can be averted by application of BC200 decoy autoantigen. As phenotype rescue was quantitatively complete, it is concluded that the observed seizure manifestations are solely caused by SLE anti-BC abs. The use of total IgG in BC200 rescue experiments in these experiments was critical: it warrants the conclusion, given the 100% rescue of the seizure phenotype by BC200 decoys, that any non–anti-BC IgG present in total IgG did not contribute to the phenotype, and therefore only anti-BC IgG did. The data indicate potential utility of BC200 decoys in autoantibody-specific therapeutic interventions with neuropsychiatric lupus patients.

## Discussion

In neurons, local protein synthesis in synapto-dendritic domains is a key mechanism in the spatiotemporal regulation of synaptic function and plasticity (Gkogkas et al, 2010; Darnell, 2011; Eom et al, 2021). Select mRNAs and components of the translational machinery are localized in postsynaptic microdomains (Tiedge & Brosius, 1996; Tübing et al, 2010; Swanger & Bassell, 2013). Translational regulators present at such sites, including the fragile X mental retardation protein (FMRP) and BC RNAs, typically control protein synthesis by repressing translation in the basal default state (Darnell & Klann, 2013; Iacoangeli & Tiedge, 2013; Lee et al, 2020). Translational control is considered necessary to avoid runaway protein synthesis. In model animals, lack of rodent BC1 RNA in neurons (either cell-wide or locally at the synapse) is associated with excessive protein synthesis and gives rise to neuronal hyperexcitability, including seizure susceptibility in vivo (Zhong et al, 2009, 2010; Briz et al, 2017; Muslimov et al, 2018).

### Human BC200 RNA: role of DTEs

A requisite precondition for local translational control mechanisms is the targeted delivery of RNAs, including mRNAs and regulatory BC RNAs, to synapto-dendritic sites of function (Doyle & Kiebler, 2011; Eom et al, 2021). In rodent BC1 RNA, a DTE in the 5′ stem-loop domain features a noncanonical GA motif with non-WC purine•purine base pairs that are required for dendritic targeting (Muslimov et al, 2006, 2011). Two potential GA motifs are contained in the 5′ stem-loop domain of non-orthologous human BC200 RNA.

We report here that dendritic delivery of human BC200 RNA is dependent on apical GA motif GA1 but not on basal GA2. The tandem noncanonical GA1 base-pairing type A•A/G•A is also found in the targeting-determinant 5′ GA motif of rodent BC1 RNA. We have previously suggested (Muslimov et al, 2006) that the G•A pair in the BC1 RNA GA motif is of the sheared type, engaging in trans Hoogsteen/Sugar-Edge interactions using two inter-base hydrogen bonds of the type N3G-N6A and N2G-N7A (Leontis & Westhof, 2001; Leontis et al, 2002). We now propose that the purine base noncanonical interactions are the same in BC200 RNA motif GA1. Identical GA motif architectural arrangements in the 5′ DTEs of rodent BC1 RNA and primate BC200 RNA are remarkable as BC1 and BC200 RNAs are non-orthologous and thus phylogenetically distinct (reviewed by Iacoangeli and Tiedge [2013] and Eom et al [2021]). It is therefore concluded that GA motifs in present-day BC1 and BC200 RNAs are the results of independent but structurally and functionally convergent motif evolution in rodent and primate phylogenetic lineages, respectively.

Recent evidence suggests that dysregulation of BC RNA dendritic targeting is associated with disease (Muslimov et al, 2018, 2019). In a subset of SLE patients with neuropsychiatric manifestations, autoantibodies engage regulatory BC200 RNA. Here, we report that SLE anti-BC IgGs specifically target the BC200 RNA 5′ GA1 motif, the same motif that, in the absence of SLE autoantibodies, is interacting with transport factors hnRNP A2 and Purα. Thus, SLE anti-BC IgGs vie with hnRNP A2 and Purα for access to the BC200 RNA DTE GA1 motif. Transport factors interact with their cargoes on an intermittent on–off basis of binding and releasing. In contrast, IgG molecules often engage their antigen targets with high affinity and essentially irreversibly, resulting in immune complexes of sufficient stability to enable effective Fcγ receptor recognition which in turn is typically a precondition for subsequent antigen neutralization and elimination (Bournazos et al, 2017; Lu et al, 2018).

Interactions of NPSLE autoantibodies with human BC200 RNA were assessed using two independent experimental assays: EMSA and IP. Both approaches reveal that recognition of BC200 RNA by NPSLE abs depends on the 5′ GA1 architectural motif that is required for dendritic targeting (Figs 5 and 6). No reactivity was observed with small nuclear U6 RNA, and serum from an SLE patient without neuropsychiatric manifestation was nonreactive with any of the tested RNAs. The data suggest a high degree of specificity in the interactions of SLE abs with BC200 RNA.

Transport factors hnRNP A2 and Purα are effectively displaced from human BC200 RNA by SLE anti-BC IgGs (Fig 7). Earlier data (Muslimov et al, 2006) indicated that GA motifs may assume one of two conformations, extended or kinked. We suggest that SLE anti-BC abs bind to only one of those conformers, as a result shifting the equilibrium toward the binding conformer in an “induced fit” mechanism. It is noted that unpaired U residues in GA motif proximity may act as hinges, facilitating conformational transitions (Muslimov et al, 2006). With two such single U residues located proximal to apical BC200 RNA GA motif GA1 (Fig 3), future work will dissect their significance in interactions with SLE anti-BC abs.

Establishing SLE anti-BC ab binding determinants in BC200 RNA will be important for the development of therapeutic BC200 decoys. We plan to design minimal BC200 decoy structures that will act as high-affinity epitopes for the specific depletion of SLE anti-BC abs from the systemic circulation. BC RNAs are afforded high intrinsic stability by their 5′ and 3′ stem-loop domains with noncanonical motif content (Eom et al, 2021). In addition, in long-term EMSA experiments (Fig 7), we observed that BC200 RNA remained stably bound to, and thus protected by, SLE anti-BC IgGs over a period of more than 4 d at 37°C. No signs of RNA degradation were observed during this time. BC200 decoy stability will also be strengthened by 2′-fluoro modification which offers excellent nuclease resistance to short RNA mimics in the circulation in vivo (Li et al, 2021).

### Phenotypic rescue: therapeutic implications

With the exception of some of the autoimmune samples listed in Table 1, SLE anti-BC sera and IgGs were obtained from NPSLE patients with seizures. Seizure disorder is a well-recognized but mechanistically poorly understood manifestation in NPSLE (Cimaz & Guerrini, 2008; Hanly, 2019; Govoni & Hanly, 2020). We assessed seizure activity after i.v. injection of SLE anti-BC abs into WT mice with permeabilized BBBs (Kowal et al, 2004). Mice injected with SLE anti-BC abs, but not control-injected mice, developed acute susceptibility to sound-induced seizures. Severe seizures of the tonic-clonic type were observed in all cases with a mortality of 100% for SLE anti-BC IgGs. Expression of seizure activity was completely averted when SLE anti-BC abs were co-applied with autoantigen (i.e., BC200 RNA or 5′ derivatives, collectively called BC200 decoys). It is concluded that pathogenic SLE autoantibodies directed against BC RNAs, not however unrelated other autoantibodies potentially present in patient sera or total IgG, are causative of the observed seizure phenotype.

The rescue data suggest therapeutic utility. Current treatments of lupus typically involve application of anti-inflammatory or immunosuppressive agents which are not specific for disease-driving autoantibodies and are therefore prone to causing side effects (Prüss, 2021). In contrast, BC200 decoys would lend themselves to target-specific therapeutic interventions for patients with neuropsychiatric SLE, especially for those with seizures. BC200 decoys can be given per i.v. infusion at levels matching those of circulating SLE anti-BC200 abs, thus neutralizing those autoantibodies by forming stable immune complexes. One would expect that typically, such immune complexes will be cleared from the circulation, for example in the spleen. For this reason, it will be important that BC200 decoys be given early to patients, before significant amounts of SLE anti-BC200 abs have been able to enter the brain. Future experiments will establish optimal time courses for the application of BC200 decoys after expression or introduction of SLE anti-BC abs in mouse models. As a caveat, it is noted that some SLE patients may have impaired clearance of immune complexes or apoptotic material, a dysregulation that may itself lead to pathogenicity (Davies et al, 1992; Munoz et al, 2005; Mahajan et al, 2016).

A further caveat is exemplified by NPSLE patients 700, 1327, and 2591 of Table 1. In these cases, strong anti-BC200 reactivity is seen in CSF but not in serum. SLE anti-BC abs, having crossed a permeable BBB, may be cleared from the circulation after BBB restoration but not from CSF. Alternatively, the very high CSF/serum reactivity ratio (i.e., the antibody or IgG index) of patients 700, 1327, and 2591 may indicate that SLE autoimmune anti-BC200 IgGs are being synthesized intrathecally (Prüss, 2021). If so, BC200 decoys used for therapeutic intervention would have to be applied by intrathecal administration (Bennett et al, 2019).

Our work suggests that human BC200 RNA can become a target of NPSLE autoantibodies. Consequently, we posit that BC200 decoys will lend themselves to autoantibody-specific treatment of NPSLE seizure disorder, thus avoiding the type of adverse effects that are caused by nonspecific chronic immunosuppressive agents (Prüss, 2021). In addition to seizures, symptoms of cognitive dysfunction are often experienced by NPSLE patients (Mader et al, 2017). Future efforts will work with anti-BC200 ab-injected animals in behavioral routines that have previously been used to establish cognitive impairment in BC1 model animals (Iacoangeli et al, 2017; Muslimov et al, 2018). It is noted that neuropsychiatric manifestations in NPSLE patients may not only be determined by the nature of the autoantibodies but also by their routes of entry into the brain (Mader et al, 2017; Prüss, 2021). It is therefore possible that an SLE autoantibody with significant anti-BC reactivity will elicit seizures and cognitive dysfunction in animals with experimentally permeabilized BBBs even though phenotypic manifestations may be more complex in patients with differentially compromised BBBs.

BC200 RNA has been implicated in a number of human malignancies, with particularly high expression levels in mammary carcinoma cells (Chen et al, 1997; Iacoangeli et al, 2004, 2018; Lee et al, 2020). Care will therefore have to be taken in the development of BC200 decoys to ensure that they do not increase cancer risk. We plan to develop minimal BC200 decoys that will contain BC200 5′ structural elements that are necessary and sufficient to specify dendritic transport (and sequester SLE anti-BC abs) but lack the central and 3′ domain elements that mediate translational control. Therefore, as such decoys will only feature transport-relevant but translation-irrelevant 5′ motif elements, they are not expected to impact translational regulation and incur risks of downstream oncogenic sequelae.

In summary, our work provides support for the thesis that SLE anti-BC abs can cause neuropsychiatric phenotypic manifestations including seizures, and it indicates that such manifestations can specifically be mitigated by application of epitope-presenting BC200 decoys.

## Materials and Methods

### RNA expression constructs

We generated transcripts from the following BC RNA plasmid constructs: (1) pBCX607 to generate full-length rat BC1 RNA (Muslimov et al, 2006, 2011); (2) pUC57_BC200 to generate full-length human BC200 RNA (Muslimov et al, 2018); (3) pBC1_IL-A:WC_ to generate BC1 RNA in which the apical GA motif has been eliminated by conversion to standard WC base pairings (Muslimov et al, 2006); (4) pBC1_∆U22_ to generate BC1 RNA lacking U22 (Muslimov et al, 2006); (5) pBC200_ΔGA1_ to generate BC200 RNA in which the apical GA motif GA1 has been eliminated by conversion to standard WC base pairings (AA-to-UC exchange); (6) BC200_ΔGA2_ to generate BC200 RNA in which the basal GA motif GA2 has been eliminated by conversion to standard WC base pairings (GAGA-to-UAUC exchange); (7) BC200_ΔGA1/ΔGA2_ to generate BC200 RNA in which both GA motifs, GA1 and GA2, have been eliminated by conversion to standard WC base pairings; and (8) pSP6-U6 to generate U6 RNA (Wang et al, 2005).

Plasmids were linearized, and ^35^S- or ^32^P-labeled transcripts were generated using T3, T7, or SP6 RNA polymerase as described (Wang et al, 2005; Muslimov et al, 2006). Excess unlabeled nucleotide (matching the radiolabeled one) was added to reactions to ensure that labeled transcripts were full-length, and size, integrity, and stability of all transcripts were monitored as described (Muslimov et al, 2006, 2011).

### Human samples

SLE patients were categorized according to the modified classification criteria of the American College of Rheumatology for SLE (Hochberg, 1997) and for NPSLE (ACR, 1999). Work with human biospecimens is being reported in accordance with the BRISQ reporting guidelines. In this study, we focus mainly on NPSLE patients with seizures, one of the common neuropsychiatric manifestations in SLE (Hanly, 2019; Govoni & Hanly, 2020). For such patients, the identification code consists of a patient number preceded by the letter S (e.g., serum SLE S1 or IgG SLE S6). Samples from the following NPSLE patients were used in the work described here: SLE S1, SLE S6, SLE S13, and SLE S17. In addition, we used samples from two non-neuropsychiatric SLE patients, SLE 10 and SLE 24. Sera from these patients were devoid of anti-BC autoimmune reactivity (Muslimov et al, 2019).

At SUNY DHSU, SLE sera were obtained from patients at the Division of Rheumatology, Department of Medicine. The study was approved by the Institutional Review Board of SUNY DHSU. Informed consent was obtained from all patients. SLE samples were collected during periods of active disease, unless stated otherwise. Personnel who participated in sample collection were not informed about subsequent sample usage.

Additional control samples included such from healthy subjects (HS), from patients with rheumatoid arthritis (RA), and from patients with multiple sclerosis (MS) (Muslimov et al, 2019). Some control serum samples were obtained from Valley Biomedical and Proteogenex. IgG was purified from human subject sera using Protein A/G and Protein G affinity columns (Nab Spin Kits; Thermo Fisher Scientific) as described (Muslimov et al, 2019).

Paired serum and CSF samples from NPSLE patients were collected at the Division of Rheumatology, Department of Medicine, Queen Elizabeth II Health Sciences Center and Dalhousie University. In most cases, paired serum and CSF samples were obtained from a patient on the same day. Exceptions are noted as follows, listing Nova Scotia patient numbers and numbers of interval days separating serum from CSF collection (with a minus sign indicating that serum was collected first, otherwise CSF first): 700, 3; 1202, 1; 2267, 2; 2317, 4; 2532, - 3; 2591, - 4; 2779, - 9; 2827, - 1.

### EMSA

For EMSA analysis, RNAs were ^32^P-labeled at 3–5 × 10^6^ cpm/μl by in vitro transcription. RNAs were incubated with transport factors or IgGs, or with serum or CSF, as described below (see also Muslimov et al [2006, 2011, 2014, 2019]). Recombinant full-length hnRNP A2 was expressed (Muslimov et al, 2011, 2014) from the plasmid pET-9c (Munro et al, 1999) and recombinant full-length Purα from the plasmid pHAPur1 which contains the PURA open reading frame with a CMV promoter and an amino-terminal HA epitope tag. This plasmid was obtained from Dr. Edward Johnson, Eastern Virginia Medical School (Gallia et al, 2000; Wortman et al, 2005).

Before incubation, RNAs were heated at 70°C for 10 min and allowed to cool for 10 min at room temperature. Purified transport factors or IgGs (10 nM) were incubated with RNA in a total of 20 μl binding buffer (100 mM KCl, 3 mM MgCl_2_, 2 mM DTT, 5% glycerol, and 20 mM Hepes, pH 7.6) for 30 min at 37°C. To minimize unspecific RNA–protein interactions, heparin was added at 5 mg/ml and incubated for 10 min at room temperature. When desired, unbound RNA was digested using RNase T1 at 30°C for 10 min. RNA–protein complexes were resolved by native PAGE on 8% gels (ratio acrylamide/bisacrylamide 19:1) in 90 mM Tris-borate, pH 8.3, 15 mM MgCl_2_, at 35 mA for 2 h at room temperature.

In long-term EMSA displacement analysis, transport factors and IgGs were incubated with radiolabeled RNA as follows. The transport factor or IgG (10 nM) was preincubated with RNA in 20 μl binding buffer at 37°C for 1 h. IgG or transport factor was then added at a 1:1 molar ratio with the respective preincubated transport factor or IgG, and incubation was continued for a total of 106 h. Aliquots of the reaction mixture were taken at sample time points as indicated in the figures.

For EMSA analysis with human sera or CSF, radiolabeled BC200 RNA (3–5 × 10^6^ cpm/μl) was mixed with serum (1 μl RNA plus 2 μl undiluted serum) or CSF (1 μl RNA plus 3 μl undiluted CSF) and incubated in binding buffer (total volume 20 μl) for 1 h at 37°C. After treatment with heparin and RNase T1, RNA–protein complexes (bound RNA) and non-complexed RNA (unbound RNA, free RNA) were resolved by PAGE as described above.

EMSA analysis is uniquely suitable and powerful in the visualization and quantification of RNA–protein interactions as it lends itself to titration, competition, and stoichiometry assay designs (Ryder et al, 2008). For an RNA–protein complex to move through a gel matrix without dissociating, binding affinities have to be relatively high. As a result, EMSA is particularly suited for the analysis of high-affinity RNA–protein complexes, whereas weaker interactions, including unspecific ones, typically result in complex dissociation during migration through the gel (Ryder et al, 2008).

### Immunoprecipitation (IP)

IP assays were performed with serum SLE S6 (from an NPSLE patient with seizures) and serum SLE 24 (from an SLE patient without neuropsychiatric involvement). Three ^32^P-labeled RNAs were used for incubation with the above sera: WT BC200 RNA, GA1-mutant BC200 RNA, and small nuclear U6 RNA. RNAs were in vitro–transcribed as described above. Dynabeads Protein G (10003D; Invitrogen) were used for serum IgG mobilization (So et al, 2016). Beads were washed five times with wash buffer (10 mM Tris–HCl, pH 7.4, 2.5 mM MgCl_2_, 100 mM NaCl, 0.1% NP40) and incubated for 2 h at 4°C with 2.5 μl of serum. Beads were again washed five times and subsequently incubated with ^32^P-labeled RNAs at 4°C overnight. Beads were again washed five times and treated with proteinase K (1 mg/ml) in proteinase K buffer (10 m M EDTA, 100 mM Tris–HCl, pH 7.5, 300 mM NaCl, 2% SDS), following which RNAs were phenol-chloroform–extracted and ethanol-precipitated. Purified RNAs were resolved on 8% polyacrylamide gels.

In a second type of IP assay, beads were washed five times after incubation with serum and subsequently incubated with 3 μg of total RNA prepared from MCF-7 cells. MCF-7 cells are breast cancer cells expressing high levels of BC200 RNA (Iacoangeli et al, 2018). After five washes and treatment with proteinase K (1 mg/ml), RNA was phenol-chloroform–extracted and ethanol-precipitated. Purified RNAs (along with input RNAs as positive controls) were analyzed by RT–PCR.

The reverse-transcription step was performed as follows, using random hexamer primers and SuperScript III (Thermo Fisher Scientific): 5 min at 25°C, 1 h at 50°C, and 15 min at 70°C. PCR amplification reactions were carried out in a final volume of 25 μl using 10 U of Platinum Taq DNA Polymerase (Invitrogen). PCR amplification conditions were as follows: 1 cycle of 1 min at 94°C, 45 s at 57°C, 1 min at 72°C, followed by 37 cycles of 30 s at 94°C, 45 s at 57°C, 1 min at 72°C, and a final cycle of 1 min at 94°C, 45 s at 57°C, and 15 min at 72°C. Primers for the amplification of BC200 RNA were as follows (expected PCR product size 98 nucleotides):

BC200 forward: 5′-CCTGGGCAATATAGCGAGAC-3′
BC200 reverse: 5′-GGGTTGTTGCTTTGAGGGA-3′

Primers for the amplification of U6 RNA were as follows (expected PCR product size 89 nucleotides):

U6 forward: 5′-GCTTCGGCAGCACATATACTAAAAT-3′
U6 reverse: 5′-CGCTTCACGAATTTGCGTGTCAT-3′

PCR products were resolved on 1% agarose gels and visualized by ethidium bromide staining. Parts of the samples were used for protein analysis to verify IgG binding to the beads. In this case, after the last wash, beads were suspended in sample buffer (blue loading buffer, 56036S; Cell Signaling) and heated at 95°C for 5 min. Protein samples were resolved by SDS–PAGE and subjected to Coomassie staining for analysis.

### Cell culture and microinjection

We maintained sympathetic neurons in low-density primary cultures as described (Muslimov et al, 2006, 2011, 2014). E19 – E20 Sprague–Dawley rat embryos (of either sex) were the source of superior cervical ganglia from which neurons were prepared. Neurons were grown on glass coverslips pre-coated with 100 μg/ml filter-sterilized poly-D-lysine (Sigma-Aldrich). Culture media were composed as follows: 50% (vol/vol) of Ham’s F12 and DMEM (Life Technologies), BSA (500 μg/ml; Calbiochem), bovine insulin (10 μg/ml; Sigma-Aldrich), rat transferrin (20 μg/ml; Jackson ImmunoResearch Laboratories), L-glutamine (20 μg/ml; Life Technologies), sodium selenite (5 ng/ml; Sigma-Aldrich), and nerve growth factor (β-NGF, 100 ng/ml; Harlan Bioproducts for Science). Basement membrane extract (Matrigel, 100 μg/ml; Collaborative Biomedical Products) was added on the third day in vitro to induce dendritic growth. To minimize proliferation of non-neuronal cells, cytosine arabinofuranoside (Ara-C, 2 μM; Sigma-Aldrich) was added on the second and fifth days after plating.

Purα siRNA treatment was performed as previously described (Eom et al, 2014). Purα siRNA was directed at a region containing the translational start site of Purα mRNA (Bergemann et al, 1992; Gallia et al, 2000). Purα siRNA and NC siRNA were obtained from Dharmacon.

We microinjected sympathetic neurons in primary culture with RNA as follows (Muslimov et al, 2006, 2011, 2014). RNAs were ^35^S-radiolabeled (∼5 × 10^6^ cpm/μl) by in vitro transcription. 10–20 fl was injected into a cell’s perikaryal region per pulse. To monitor the injection process and to ascertain that experimental manipulations did not cause alterations in neuronal cell morphology (e.g., dendritic extent and arborization), we coinjected Lucifer yellow (LY, 0.4%) in all RNA injections of cultured neurons (Muslimov et al, 2006, 2011, 2014). Three methods were used to ascertain transcript stability: (i) preinjection by PAGE, (ii) postinjection by measuring average integrated total signal intensities per injected cell, and (iii) incubation with brain extract (Muslimov et al, 2011, 2014).

For reasons described in previous communications (Muslimov et al, 2006, 2011, 2014), we continue to rely on microinjection of radiolabeled transcripts to introduce RNAs into neurons. The reasons are twofold. (i) We found that microinjection affords exquisite control of amounts of RNA introduced. Therefore, and because of the high sensitivity of radiolabel detection, microinjection is able to introduce RNAs at levels lower than those of respective endogenous RNAs (Muslimov et al, 2006, 2011, 2014). (ii) Architectural GA motifs, central to dendritic targeting of BC RNAs, are quite intolerant of nucleotide substitutions and of introduced side chains that may disrupt motif structure and, as a result, interfere with factor binding and dendritic targeting (Goody et al, 2004; Muslimov et al, 2011, 2014). We therefore remain committed to using radiolabeled transcripts in RNA transport studies.

For emulsion autoradiography, dried coverslips were mounted, cell side up, on microscope slides and dipped in NTB2 emulsion (Kodak). Cells were exposed at 4°C for 7 d before photographic development (Kodak D-19 developer, 50% strength; Kodak Rapid-Fix) as described (Muslimov et al, 1998, 2011).

### Microscopy

A Nikon Microphot-FXA microscope was used for dark-field and phase-contrast microscopy (Muslimov et al, 2018, 2019). Photomicrographs were taken with a Digital Sight DS-Fi1 five-megapixel charge-coupled device (CCD) camera (Nikon). Photographs were taken of fixed specimens (cultured neurons on coverslips mounted as described above) at room temperature using the following objectives: (1) Plan Fluor 10×/0.30, 160/0.17; (2) PhC Plan 20×/0.50, DL 160/0.17; (3) Plan 20×/0.50, DIC 160/0.17; and (4) Ph3 DL Plan 40×/0.65, 160/0.17. To evaluate transport of injected RNAs, silver grains were measured along dendritic shafts at 50-μm interval points, up to 250 μm (Muslimov et al, 2006, 2011). Photoshop and Illustrator softwares (Adobe Systems) were used to generate illustrations and to arrange final figures. All experiments were performed at least in quadruplicate or as noted in figure legends. For statistical analysis, see the below section Quantitative and Statistical Analyses.

### Animals

Work with vertebrate animals was approved by the SUNY DHSU Institutional Animal Care and Use Committee (IACUC) and is reported in accordance with ARRIVE guidelines. Animals were housed in the Division of Comparative Medicine (DCM) of SUNY DHSU.

Animals of both sexes were used. I.v. injections of SLE anti-BC sera or IgGs, or control sera or IgGs, were performed with WT mice and BC1 KO mice. In our previous work (Muslimov et al, 2019), i.v. injections were performed with BALB/c mice as this strain has been used in an earlier publication that established the technique (Kowal et al, 2006). In the present work, i.v. injections were performed to address the question whether NPSLE anti-BC abs would cause hyperexcitability manifesting in seizure susceptibility. Hyperexcitability caused by neuronal translational dysregulation has in the past typically been investigated using mice of mixed background strains, usually including a C57BL/6J component (e.g., Chuang et al [2005]). For cross-comparability, we have in our work with BC1 model animals used a C57BL/6J x 129X1/SvJ mixed background strain (Zhong et al, 2009, 2010; Muslimov et al, 2018). For the same reason, we used animals of the C57BL/6J x 129X1/SvJ strain in our current analysis of seizure susceptibility caused by SLE autoantibodies. WT mice of various background strains, including the C57BL/6J x 129X1/SvJ strain used by us, are typically not susceptible to sound-induced seizures. In an exception, however, WT mice of the FVB strain are seizure-prone (albeit at low levels; Yan et al, 2004) which makes them unsuitable for seizure susceptibility experiments. Mice of a pure C57BL/6J background have been reported to be impaired in neuronal translation as a result of a mutation in a brain-specific tRNA gene (Darnell, 2014; Ishimura et al, 2014).

BC1 model mice and WT mice injected with SLE anti-BC abs are sensitive to auditory stimulation and may undergo seizures upon exposure to loud sounds. These animals are therefore housed in a designated “quiet room” (room 9-035B) where such exposure is minimized. The room is separated from other animal colonies (including a macaque colony) and is physically isolated by dual-door entry. Access to this room is restricted to authorized personnel trained in the husbandry of these animals, and animal occupancy is limited to a single-PI IACUC protocol. The DCM quiet-room requirement (i.e., minimal environmental noise) is a formal component of the approved IACUC protocol. Food (standard pellets) and water were available ad libitum. All animals were housed in compliant cages.

### Injection of mice with NPSLE and control sera and antibodies

Sera and IgGs, obtained from NPSLE patients SLE S1, S6, and S13, were i.v. injected into mice as described (Kowal et al, 2006) using the retro-orbital route (Yardeni et al, 2011; Schoch et al, 2014). Controls included sera and IgGs from a healthy subject (HS4), from patients with non-SLE autoimmune disorders (rheumatoid arthritis, patient RA1, and multiple sclerosis, patient MS1), and from a non-NP SLE patient (patient SLE 10). Sera were injected twice, on postnatal day 14 (P14, 15 μl, right side) and on day P15 (15 μl, left side). Purified IgG fractions (0.25 mg/ml in 15 μl saline) were injected once on day P15. 15 min after each serum or IgG injection, LPS (3 mg/kg) was injected i.p. for permeabilization of the BBB. A further dose of LPS was administered i.p. on day P17. Animals were scored for seizure parameters (including seizure rate and mortality, see below) on day P19. The injection schedule was designed to ensure clearance of preexisting endogenous BC1 RNA from dendrites without risking clearance of injected antibodies from circulation or brain (Muslimov et al, 2011; Schoch et al, 2014).

Full-length BC200 RNA and the BC200 5′ domain were used as decoys. BC200 decoys were i.v. coinjected with SLE sera or purified IgG. The molar ratio of IgG:decoy was 1:1.

### Seizure susceptibility

Seizure susceptibility that results from dysregulated translational control in neurons has in the past typically been analyzed using auditory stimulation (Musumeci et al, 2000, 2007; Yan et al, 2004; Osterweil et al, 2010). For this reason, we have used this experimental paradigm in our previous work with BC1 model animals (Zhong et al, 2009, 2010; Muslimov et al, 2018), and we continue to do so in the current work with WT animals injected with SLE anti-BC abs. However, incidental observations indicate that other types of sensory stimulation (i.e., olfactory, visual) can also cause seizure susceptibility in such animals.

Injected animals—females and males were used—were examined singly in a plastic cage equipped with a 120-dB personal alarm device (TBO-Tech) mounted under the cage cover. Auditory stimulation was applied for 15 min. End-point experimenters of in vivo seizure susceptibility tests were not cognizant of animals’ injection protocols (e.g., NPSLE versus non-SLE or non-NP SLE or NPSLE plus decoys). All in vivo seizure susceptibility tests were videotaped, and all videos were independently viewed by staff without knowledge of the animals’ experimental histories. The following parameters were scored: time to onset of wild running, time to onset of seizures, percentage of animals undergoing tonic-clonic seizures, time to death, and percent mortality during seizures. Whenever scored parameters did not significantly differ between groups of animals (e.g., seizure rates between females and males), they were analyzed jointly.

### Quantitative and statistical analyses

Sympathetic neurons in primary culture were used for RNA dendritic transport experiments. The overall experimental design followed previously published protocols (Muslimov et al, 2006, 2011, 2014). To ensure that measured RNA transport parameters were not impacted by varying amounts of injected RNA, we microinjected each RNA at a range of concentrations that spanned at least one order of magnitude. To quantify dendritic localization, we proceeded as follows.

Silver grain densities were established along the dendritic extent at 50-μm interval sample points, up to 250 μm. A 0-μm sample point was defined at the base of a dendrite and was assigned a relative signal intensity of 100%. Statistical analysis was performed using one-way ANOVA followed by Dunnett’s test for multiple comparisons (comparison of signal intensities at all sample points) (Muslimov et al, 2006, 2011, 2014). Data points and error bars in this study are given as mean ± SEM unless noted otherwise.

Naive WT mice were i.v. injected with SLE anti-BC sera or IgGs from NPSLE patients with seizures or with control sera or IgGs. We asked if SLE anti-BC abs so injected would give rise to seizure susceptibility. Statistical significance of seizure data was examined using Fisher’s exact test, analyzing seizure versus no-seizure and death versus survival outcomes of animals injected with NPSLE sera or IgGs, with NPSLE sera or IgGs in the presence of BC200 decoys, with non-NP SLE sera, or with non-SLE sera or IgGs. Confidence intervals were calculated using the Agresti-Coull method (Agresti & Coull, 1998).

Quantitative and statistical analyses were performed using Prism (GraphPad), SPSS Statistics (IBM, RRID:SCR_002865), and the R Project for Statistical Computing (The R Foundation). Significance levels are identified as follows: *P < 0.05, **P < 0.01, ***P < 0.001.

## References

[bib1] ACR (1999) The American College of Rheumatology nomenclature and case definitions for neuropsychiatric lupus syndromes. Arthritis Rheum 42: 599–608. 10.1002/1529-0131(199904)42:4<599::aid-anr2>3.0.co;2-f 10211873

[bib2] Agresti A, Coull BA (1998) Approximate is better than “exact” for interval estimation of binomial proportions. American Statistician 52: 119–126. 10.2307/2685469

[bib3] Bennett CF, Krainer AR, Cleveland DW (2019) Antisense oligonucleotide therapies for neurodegenerative diseases. Annu Rev Neurosci 42: 385–406. 10.1146/annurev-neuro-070918-050501 31283897 PMC7427431

[bib4] Bergemann AD, Ma ZW, Johnson EM (1992) Sequence of cDNA comprising the human pur gene and sequence-specific single-stranded-DNA-binding properties of the encoded protein. Mol Cell Biol 12: 5673–5682. 10.1128/mcb.12.12.5673-5682.1992 1448097 PMC360507

[bib5] Booy EP, McRae EK, Ezzati P, Choi T, Gussakovsky D, McKenna SA (2018) Comprehensive analysis of the BC200 ribonucleoprotein reveals a reciprocal regulatory function with CSDE1/UNR. Nucleic Acids Res 46: 11575–11591. 10.1093/nar/gky860 30247708 PMC6265466

[bib6] Booy EP, McRae EK, Howard R, Deo SR, Ariyo EO, Dzananovic E, Meier M, Stetefeld J, McKenna SA (2016) RNA helicase associated with AU-rich element (RHAU/DHX36) interacts with the 3’-tail of the long non-coding RNA BC200 (BCYRN1). J Biol Chem 291: 5355–5372. 10.1074/jbc.m115.711499 26740632 PMC4777866

[bib7] Bournazos S, Wang TT, Dahan R, Maamary J, Ravetch JV (2017) Signaling by antibodies: Recent progress. Annu Rev Immunol 35: 285–311. 10.1146/annurev-immunol-051116-052433 28446061 PMC5613280

[bib8] Briz V, Restivo L, Pasciuto E, Juczewski K, Mercaldo V, Lo AC, Baatsen P, Gounko NV, Borreca A, Girardi T, (2017) The non-coding RNA BC1 regulates experience-dependent structural plasticity and learning. Nat Commun 8: 293. 10.1038/s41467-017-00311-2 28819097 PMC5561022

[bib9] Chao JA, Patskovsky Y, Patel V, Levy M, Almo SC, Singer RH (2010) ZBP1 recognition of beta-actin zipcode induces RNA looping. Genes Dev 24: 148–158. 10.1101/gad.1862910 20080952 PMC2807350

[bib10] Chen W, Böcker W, Brosius J, Tiedge H (1997) Expression of neural BC200 RNA in human tumours. J Pathol 183: 345–351. 10.1002/(sici)1096-9896(199711)183:3<345::aid-path930>3.0.co;2-8 9422992

[bib11] Chuang SC, Zhao W, Bauchwitz R, Yan Q, Bianchi R, Wong RK (2005) Prolonged epileptiform discharges induced by altered group I metabotropic glutamate receptor-mediated synaptic responses in hippocampal slices of a fragile X mouse model. J Neurosci 25: 8048–8055. 10.1523/jneurosci.1777-05.2005 16135762 PMC6725444

[bib12] Cimaz R, Guerrini R (2008) Epilepsy in lupus. Lupus 17: 777–779. 10.1177/0961203308094362 18755857

[bib13] Congdon EE, Gu J, Sait HB, Sigurdsson EM (2013) Antibody uptake into neurons occurs primarily via clathrin-dependent Fcγ receptor endocytosis and is a prerequisite for acute tau protein clearance. J Biol Chem 288: 35452–35465. 10.1074/jbc.m113.491001 24163366 PMC3853292

[bib14] Darnell JC (2011) Defects in translational regulation contributing to human cognitive and behavioral disease. Curr Opin Genet Dev 21: 465–473. 10.1016/j.gde.2011.05.002 21764293 PMC3166213

[bib15] Darnell JC, Klann E (2013) The translation of translational control by FMRP: Therapeutic targets for FXS. Nat Neurosci 16: 1530–1536. 10.1038/nn.3379 23584741 PMC3999698

[bib16] Darnell JC (2014) Ribosome rescue and neurodegeneration. Science 345: 378–379. 10.1126/science.1257193 25061188

[bib17] Davies KA, Peters AM, Beynon HL, Walport MJ (1992) Immune complex processing in patients with systemic lupus erythematosus. In vivo imaging and clearance studies. J Clin Invest 90: 2075–2083. 10.1172/jci116090 1430231 PMC443274

[bib18] DeChiara TM, Brosius J (1987) Neural BC1 RNA: cDNA clones reveal nonrepetitive sequence content. Proc Natl Acad Sci U S A 84: 2624–2628. 10.1073/pnas.84.9.2624 2437583 PMC304710

[bib19] Douglas JN, Gardner LA, Levin MC (2013) Antibodies to an intracellular antigen penetrate neuronal cells and cause deleterious effects. J Clin Cell Immunol 4: 1–7. 10.4172/2155-9899.1000134

[bib20] Doyle M, Kiebler MA (2011) Mechanisms of dendritic mRNA transport and its role in synaptic tagging. EMBO J 30: 3540–3552. 10.1038/emboj.2011.278 21878995 PMC3181491

[bib21] Elkon K, Casali P (2008) Nature and functions of autoantibodies. Nat Clin Pract Rheumatol 4: 491–498. 10.1038/ncprheum0895 18756274 PMC2703183

[bib22] Eom T, Berardi V, Zhong J, Risuleo G, Tiedge H (2011) Dual nature of translational control by regulatory BC RNAs. Mol Cell Biol 31: 4538–4549. 10.1128/mcb.05885-11 21930783 PMC3209249

[bib23] Eom T, Muslimov IA, Iacoangeli A, Tiedge H (2021) Dendritic targeting and regulatory RNA control of local neuronal translation. In The Oxford Handbook of Neuronal Protein Synthesis, Sossin W (ed), pp 105–129. New York: Oxford University Press. Published online at oxfordhandbooks.com.

[bib24] Eom T, Muslimov IA, Tsokas P, Berardi V, Zhong J, Sacktor TC, Tiedge H (2014) Neuronal BC RNAs cooperate with eIF4B to mediate activity-dependent translational control. J Cell Biol 207: 237–252. 10.1083/jcb.201401005 25332164 PMC4210447

[bib25] Gallia GL, Johnson EM, Khalili K (2000) Survey and summary: Puralpha: A multifunctional single-stranded DNA- and RNA-binding protein. Nucleic Acids Res 28: 3197–3205. 10.1093/nar/28.17.3197 10954586 PMC110688

[bib26] Geis C, Weishaupt A, Hallermann S, Grunewald B, Wessig C, Wultsch T, Reif A, Byts N, Beck M, Jablonka S, (2010) Stiff person syndrome-associated autoantibodies to amphiphysin mediate reduced GABAergic inhibition. Brain 133: 3166–3180. 10.1093/brain/awq253 20884644

[bib27] Gkogkas C, Sonenberg N, Costa-Mattioli M (2010) Translational control mechanisms in long-lasting synaptic plasticity and memory. J Biol Chem 285: 31913–31917. 10.1074/jbc.r110.154476 20693284 PMC2952191

[bib28] Goody TA, Melcher SE, Norman DG, Lilley DM (2004) The kink-turn motif in RNA is dimorphic, and metal ion-dependent. RNA 10: 254–264. 10.1261/rna.5176604 14730024 PMC1370537

[bib29] Govoni M, Hanly JG (2020) The management of neuropsychiatric lupus in the 21st century: Still so many unmet needs? Rheumatology (Oxford) 59: v52–v62. 10.1093/rheumatology/keaa404 33280014 PMC7719041

[bib30] Hanly JG (2019) Overview of Neuropsychiatric SLE: Current Status and Future Directions. Atlanta, GA: ACR/ARP Annual Meeting.

[bib31] Hochberg MC (1997) Updating the American College of Rheumatology revised criteria for the classification of systemic lupus erythematosus. Arthritis Rheum 40: 1725. 10.1002/art.1780400928 9324032

[bib32] Iacoangeli A, Adzovic L, Chen EQ, Latif Cattie R, Soff GA, Tiedge H (2018) Regulatory BC200 RNA in peripheral blood of patients with invasive breast cancer. J Investig Med 66: 1055–1063. 10.1136/jim-2018-000717 PMC6158080 29967012

[bib33] Iacoangeli A, Dosunmu A, Eom T, Stefanov DG, Tiedge H (2017) Regulatory BC1 RNA in cognitive control. Learn Mem 24: 267–277. 10.1101/lm.045427.117 28620074 PMC5473108

[bib34] Iacoangeli A, Lin Y, Morley EJ, Muslimov IA, Bianchi R, Reilly J, Weedon J, Diallo R, Böcker W, Tiedge H (2004) BC200 RNA in invasive and preinvasive breast cancer. Carcinogenesis 25: 2125–2133. 10.1093/carcin/bgh228 15240511

[bib35] Iacoangeli A, Tiedge H (2013) Translational control at the synapse: Role of RNA regulators. Trends Biochem Sci 38: 47–55. 10.1016/j.tibs.2012.11.001 23218750 PMC3530003

[bib36] Ishimura R, Nagy G, Dotu I, Zhou H, Yang XL, Schimmel P, Senju S, Nishimura Y, Chuang JH, Ackerman SL (2014) Ribosome stalling induced by mutation of a CNS-specific tRNA causes neurodegeneration. Science 345: 455–459. 10.1126/science.1249749 25061210 PMC4281038

[bib37] Job C, Eberwine J (2001) Localization and translation of mRNA in dentrites and axons. Nat Rev Neurosci 2: 889–898. 10.1038/35104069 11733796

[bib38] Jung E, Lee J, Hong HJ, Park I, Lee Y (2014) RNA recognition by a human antibody against brain cytoplasmic 200 RNA. RNA 20: 805–814. 10.1261/rna.040899.113 24759090 PMC4024635

[bib39] Kazim SF, Chuang SC, Zhao W, Wong RKS, Bianchi R, Iqbal K (2017) Early-onset network hyperexcitability in presymptomatic alzheimer’s disease transgenic mice is suppressed by passive immunization with anti-human APP/aβ antibody and by mGluR5 blockade. Front Aging Neurosci 9: 71. 10.3389/fnagi.2017.00071 28392767 PMC5364175

[bib40] Khanam T, Rozhdestvensky TS, Bundman M, Galiveti CR, Handel S, Sukonina V, Jordan U, Brosius J, Skryabin BV (2007) Two primate-specific small non-protein-coding RNAs in transgenic mice: Neuronal expression, subcellular localization and binding partners. Nucleic Acids Res 35: 529–539. 10.1093/nar/gkl1082 17175535 PMC1802616

[bib41] Kowal C, Degiorgio LA, Lee JY, Edgar MA, Huerta PT, Volpe BT, Diamond B (2006) Human lupus autoantibodies against NMDA receptors mediate cognitive impairment. Proc Natl Acad Sci U S A 103: 19854–19859. 10.1073/pnas.0608397104 17170137 PMC1702320

[bib42] Kowal C, DeGiorgio LA, Nakaoka T, Hetherington H, Huerta PT, Diamond B, Volpe BT (2004) Cognition and immunity. Immunity 21: 179–188. 10.1016/j.immuni.2004.07.011 15308099

[bib43] Lee Y, Lee HS, Kim M, Shin H (2020) Brain cytoplasmic RNAs in neurons: From biosynthesis to function. Biomolecules 10: 313. 10.3390/biom10020313 PMC7072442 32079202

[bib44] Leontis NB, Stombaugh J, Westhof E (2002) The non-Watson-Crick base pairs and their associated isostericity matrices. Nucleic Acids Res 30: 3497–3531. 10.1093/nar/gkf481 12177293 PMC134247

[bib45] Leontis NB, Westhof E (2001) Geometric nomenclature and classification of RNA base pairs. RNA 7: 499–512. 10.1017/s1355838201002515 11345429 PMC1370104

[bib46] Li Y, Tan Z, Zhang Y, Zhang Z, Hu Q, Liang K, Jun Y, Ye Y, Li YC, Li C, (2021) A noncoding RNA modulator potentiates phenylalanine metabolism in mice. Science 373: 662–673. 10.1126/science.aba4991 34353949 PMC9714245

[bib47] Lin D, Pestova TV, Hellen CUT, Tiedge H (2008) Translational control by a small RNA: Dendritic BC1 RNA targets the eukaryotic initiation factor 4A helicase mechanism. Mol Cell Biol 28: 3008–3019. 10.1128/mcb.01800-07 18316401 PMC2293081

[bib48] Lin Y, Brosius J, Tiedge H (2001) Neuronal BC1 RNA: Co-expression with growth-associated protein-43 messenger RNA. Neuroscience 103: 465–479. 10.1016/s0306-4522(01)00003-3 11246161

[bib49] Lu LL, Suscovich TJ, Fortune SM, Alter G (2018) Beyond binding: Antibody effector functions in infectious diseases. Nat Rev Immunol 18: 46–61. 10.1038/nri.2017.106 29063907 PMC6369690

[bib50] Mader S, Brimberg L, Diamond B (2017) The role of brain-reactive autoantibodies in brain pathology and cognitive impairment. Front Immunol 8: 1101. 10.3389/fimmu.2017.01101 28955334 PMC5601985

[bib51] Magrys A, Anekonda T, Ren G, Adamus G (2007) The role of anti-alpha-enolase autoantibodies in pathogenicity of autoimmune-mediated retinopathy. J Clin Immunol 27: 181–192. 10.1007/s10875-006-9065-8 17235687

[bib52] Mahajan A, Herrmann M, Munoz LE (2016) Clearance deficiency and cell death pathways: A model for the pathogenesis of SLE. Front Immunol 7: 35. 10.3389/fimmu.2016.00035 26904025 PMC4745266

[bib53] Martignetti JA, Brosius J (1993a) BC200 RNA: A neural RNA polymerase III product encoded by a monomeric alu element. Proc Natl Acad Sci U S A 90: 11563–11567. 10.1073/pnas.90.24.11563 8265590 PMC48024

[bib54] Martignetti JA, Brosius J (1993b) Neural BC1 RNA as an evolutionary marker: Guinea pig remains a rodent. Proc Natl Acad Sci U S A 90: 9698–9702. 10.1073/pnas.90.20.9698 7692450 PMC47637

[bib55] McKinnon RD, Danielson P, Brow MAD, Bloom FE, Sutcliffe JG (1987) Expression of small cytoplasmic transcripts of the rat identifier element in vivo and in cultured cells. Mol Cell Biol 7: 2148–2154. 10.1128/mcb.7.6.2148-2154.1987 2439903 PMC365337

[bib56] Mladek A, Sharma P, Mitra A, Bhattacharyya D, Sponer J, Sponer JE (2009) Trans Hoogsteen/sugar edge base pairing in RNA. Structures, energies, and stabilities from quantum chemical calculations. J Phys Chem B 113: 1743–1755. 10.1021/jp808357m 19152254

[bib57] Munoz LE, Gaipl US, Franz S, Sheriff A, Voll RE, Kalden JR, Herrmann M (2005) SLE: A disease of clearance deficiency? Rheumatology (Oxford) 44: 1101–1107. 10.1093/rheumatology/keh693 15928001

[bib58] Munro TP, Magee RJ, Kidd GJ, Carson JH, Barbarese E, Smith LM, Smith R (1999) Mutational analysis of a heterogeneous nuclear ribonucleoprotein A2 response element for RNA trafficking. J Biol Chem 274: 34389–34395. 10.1074/jbc.274.48.34389 10567417

[bib59] Muslimov IA, Banker G, Brosius J, Tiedge H (1998) Activity-dependent regulation of dendritic BC1 RNA in hippocampal neurons in culture. J Cell Biol 141: 1601–1611. 10.1083/jcb.141.7.1601 9647652 PMC1828539

[bib60] Muslimov IA, Eom T, Iacoangeli A, Chuang SC, Hukema RK, Willemsen R, Stefanov DG, Wong RKS, Tiedge H (2018) BC RNA mislocalization in the fragile X premutation. eNeuro 5: ENEURO.0091–18.2018. 10.1523/eneuro.0091-18.2018 PMC5952321 29766042

[bib61] Muslimov IA, Iacoangeli A, Brosius J, Tiedge H (2006) Spatial codes in dendritic BC1 RNA. J Cell Biol 175: 427–439. 10.1083/jcb.200607008 17074884 PMC1808587

[bib62] Muslimov IA, Iacoangeli A, Eom T, Ruiz A, Lee M, Stephenson S, Ginzler EM, Tiedge H (2019) Neuronal BC RNA transport impairments caused by systemic lupus erythematosus autoantibodies. J Neurosci 39: 7759–7777. 10.1523/jneurosci.1657-18.2019 31405929 PMC6764197

[bib63] Muslimov IA, Patel MV, Rose A, Tiedge H (2011) Spatial code recognition in neuronal RNA targeting: Role of RNA-hnRNP A2 interactions. J Cell Biol 194: 441–457. 10.1083/jcb.201010027 21807882 PMC3153643

[bib64] Muslimov IA, Tuzhilin A, Tang TH, Wong RK, Bianchi R, Tiedge H (2014) Interactions of noncanonical motifs with hnRNP A2 promote activity-dependent RNA transport in neurons. J Cell Biol 205: 493–510. 10.1083/jcb.201310045 24841565 PMC4033767

[bib65] Musumeci SA, Bosco P, Calabrese G, Bakker C, Sarro GB, Elia M, Ferri R, Oostra BA (2000) Audiogenic seizures susceptibility in transgenic mice with fragile X syndrome. Epilepsia 41: 19–23. 10.1111/j.1528-1157.2000.tb01499.x 10643918

[bib66] Musumeci SA, Calabrese G, Bonaccorso CM, D’Antoni S, Brouwer JR, Bakker CE, Elia M, Ferri R, Nelson DL, Oostra BA, (2007) Audiogenic seizure susceptibility is reduced in fragile X knockout mice after introduction of FMR1 transgenes. Exp Neurol 203: 233–240. 10.1016/j.expneurol.2006.08.007 17007840

[bib67] Osterweil EK, Krueger DD, Reinhold K, Bear MF (2010) Hypersensitivity to mGluR5 and ERK1/2 leads to excessive protein synthesis in the hippocampus of a mouse model of fragile X syndrome. J Neurosci 30: 15616–15627. 10.1523/jneurosci.3888-10.2010 21084617 PMC3400430

[bib68] Prüss H (2021) Autoantibodies in neurological disease. Nat Rev Immunol 21: 798–813. 10.1038/s41577-021-00543-w 33976421 PMC8111372

[bib69] Rhodes DA, Isenberg DA (2017) TRIM21 and the function of antibodies inside cells. Trends Immunol 38: 916–926. 10.1016/j.it.2017.07.005 28807517

[bib70] Rozhdestvensky TS, Kopylov AM, Brosius J, Hüttenhofer A (2001) Neuronal BC1 RNA structure: Evolutionary conversion of a tRNA^Ala^ domain into an extended stem-loop structure. RNA 7: 722–730. 10.1017/s1355838201002485 11350036 PMC1370124

[bib71] Ryder SP, Recht MI, Williamson JR (2008) Quantitative analysis of protein-RNA interactions by gel mobility shift. Methods Mol Biol 488: 99–115. 10.1007/978-1-60327-475-3_7 18982286 PMC2928675

[bib72] Schoch A, Thorey IS, Engert J, Winter G, Emrich T (2014) Comparison of the lateral tail vein and the retro-orbital venous sinus routes of antibody administration in pharmacokinetic studies. Lab Anim (NY) 43: 95–99. 10.1038/laban.481 24552915

[bib73] Skryabin BV, Kremerskothen J, Vassilacopoulou D, Disotell TR, Kapitonov VV, Jurka J, Brosius J (1998) The BC200 RNA gene and its neural expression are conserved in Anthropoidea (Primates). J Mol Evol 47: 677–685. 10.1007/pl00006426 9847409

[bib74] Skryabin BV, Sukonina V, Jordan U, Lewejohann L, Sachser N, Muslimov I, Tiedge H, Brosius J (2003) Neuronal untranslated BC1 RNA: Targeted gene elimination in mice. Mol Cell Biol 23: 6435–6441. 10.1128/mcb.23.18.6435-6441.2003 12944471 PMC193692

[bib75] So BR, Wan L, Zhang Z, Li P, Babiash E, Duan J, Younis I, Dreyfuss G (2016) A U1 snRNP-specific assembly pathway reveals the SMN complex as a versatile hub for RNP exchange. Nat Struct Mol Biol 23: 225–230. 10.1038/nsmb.3167 26828962 PMC4834709

[bib76] Sosińska-Zawierucha P, Zawierucha P, Bręborowicz A, Barciszewski J (2018) Prediction of secondary and tertiary structures of human BC200 RNA (BCYRN1) based on experimental and bioinformatic cross-validation. Biochem J 475: 2727–2748. 10.1042/bcj20180239 30072491

[bib77] Sutcliffe JG, Milner RJ, Gottesfeld JM, Reynolds W (1984) Control of neuronal gene expression. Science 225: 1308–1315. 10.1126/science.6474179 6474179

[bib78] Swanger SA, Bassell GJ (2013) Dendritic protein synthesis in the normal and diseased brain. Neuroscience 232: 106–127. 10.1016/j.neuroscience.2012.12.003 23262237 PMC4502914

[bib79] Tiedge H, Brosius J (1996) Translational machinery in dendrites of hippocampal neurons in culture. J Neurosci 16: 7171–7181. 10.1523/jneurosci.16-22-07171.1996 8929426 PMC6578937

[bib80] Tiedge H, Chen W, Brosius J (1993) Primary structure, neural-specific expression, and dendritic location of human BC200 RNA. J Neurosci 13: 2382–2390. 10.1523/jneurosci.13-06-02382.1993 7684772 PMC6576500

[bib81] Tiedge H, Fremeau RT, Jr, Weinstock PH, Arancio O, Brosius J (1991) Dendritic location of neural BC1 RNA. Proc Natl Acad Sci U S A 88: 2093–2097. 10.1073/pnas.88.6.2093 1706516 PMC51175

[bib82] Tübing F, Vendra G, Mikl M, Macchi P, Thomas S, Kiebler MA (2010) Dendritically localized transcripts are sorted into distinct ribonucleoprotein particles that display fast directional motility along dendrites of hippocampal neurons. J Neurosci 30: 4160–4170. 10.1523/jneurosci.3537-09.2010 20237286 PMC6632293

[bib83] Wang H, Iacoangeli A, Lin D, Williams K, Denman RB, Hellen CUT, Tiedge H (2005) Dendritic BC1 RNA in translational control mechanisms. J Cell Biol 171: 811–821. 10.1083/jcb.200506006 16330711 PMC1828541

[bib84] Wortman MJ, Johnson EM, Bergemann AD (2005) Mechanism of DNA binding and localized strand separation by Purα and comparison with Pur family member, Purβ. Biochim Biophys Acta 1743: 64–78. 10.1016/j.bbamcr.2004.08.010 15777841

[bib85] Yan QJ, Asafo-Adjei PK, Arnold HM, Brown RE, Bauchwitz RP (2004) A phenotypic and molecular characterization of the fmr1-tm1Cgr fragile X mouse. Genes Brain Behav 3: 337–359. 10.1111/j.1601-183x.2004.00087.x 15544577

[bib86] Yardeni T, Eckhaus M, Morris HD, Huizing M, Hoogstraten-Miller S (2011) Retro-orbital injections in mice. Lab Anim (NY) 40: 155–160. 10.1038/laban0511-155 21508954 PMC3158461

[bib87] Zhong J, Chuang SC, Bianchi R, Zhao W, Lee H, Fenton AA, Wong RKS, Tiedge H (2009) BC1 regulation of metabotropic glutamate receptor-mediated neuronal excitability. J Neurosci 29: 9977–9986. 10.1523/jneurosci.3893-08.2009 19675232 PMC2866649

[bib88] Zhong J, Chuang SC, Bianchi R, Zhao W, Paul G, Thakkar P, Liu D, Fenton AA, Wong RKS, Tiedge H (2010) Regulatory BC1 RNA and the fragile X mental retardation protein: Convergent functionality in brain. PLoS One 5: e15509. 10.1371/journal.pone.0015509 21124905 PMC2990754

